# An engineered human Fc domain that behaves like a pH-toggle switch for ultra-long circulation persistence

**DOI:** 10.1038/s41467-019-13108-2

**Published:** 2019-11-06

**Authors:** Chang-Han Lee, Tae Hyun Kang, Ophélie Godon, Makiko Watanabe, George Delidakis, Caitlin M. Gillis, Delphine Sterlin, David Hardy, Michel Cogné, Lynn E. Macdonald, Andrew J. Murphy, Naxin Tu, Jiwon Lee, Jonathan R. McDaniel, Emily Makowski, Peter M. Tessier, Aaron S. Meyer, Pierre Bruhns, George Georgiou

**Affiliations:** 1 Department of Chemical Engineering, University of Texas at Austin, Austin, TX USA; 2 Unit of Antibodies in Therapy and Pathology, Institut Pasteur, UMR1222 INSERMF-75015, Paris, France; 3 Experimental Neuropathology Unit, Infection and Epidemiology Department, Institut Pasteur, 25, rue du Docteur Roux, 75015 Paris, France; 4 Limoges University, Limoges, France; 5 Regeneron Pharmaceuticals, Inc., Tarrytown, NY USA; 6 Thayer School of Engineering, Dartmouth College, Hanover, NH USA; 7 Department of Pharmaceutical Sciences, University of Michigan, Ann Arbor, MI USA; 8 Department of Chemical Engineering, University of Michigan, Ann Arbor, MI USA; 9 Department of Biomedical Engineering, University of Michigan, Ann Arbor, MI USA; 10 Department of Bioengineering, University of California at Los Angeles, Los Angeles, CA USA; 11 Department of Molecular Bioscience, University of Texas at Austin, Austin, TX USA; 12 Department of Biomedical Engineering, University of Texas at Austin, Austin, TX USA; 13 Present Address: Department of Applied Chemistry, Kookmin University, Seoul, Republic of Korea

**Keywords:** Genetic engineering, Protein design

## Abstract

The pharmacokinetic properties of antibodies are largely dictated by the pH-dependent binding of the IgG fragment crystallizable (Fc) domain to the human neonatal Fc receptor (hFcRn). Engineered Fc domains that confer a longer circulation half-life by virtue of more favorable pH-dependent binding to hFcRn are of great therapeutic interest. Here we developed a pH Toggle switch Fc variant containing the L309D/Q311H/N434S (DHS) substitutions, which exhibits markedly improved pharmacokinetics relative to both native IgG1 and widely used half-life extension variants, both in conventional hFcRn transgenic mice and in new knock-in mouse strains. engineered specifically to recapitulate all the key processes relevant to human antibody persistence in circulation, namely: (i) physiological expression of hFcRn, (ii) the impact of hFcγRs on antibody clearance and (iii) the role of competing endogenous IgG. DHS-IgG retains intact effector functions, which are important for the clearance of target pathogenic cells and also has favorable developability.

## Abstract (web summary)

Lee *et al*. report an engineered IgG1 Fc domain that behaves like an hFcRn binding pH toggle switch. The authors show that this new half-life extension Fc domain confers improved pharmacokinetics in new humanized knock-in mouse strains that recapitulate the key processes for antibody persistence in circulation.

## Introduction

IgG isotype antibodies avoid clearance by endolysosomal degradation by virtue of their pH-dependent binding to the neonatal Fc receptor (FcRn), which is expressed by cells in nearly every organ in mammals^1–3^. The FcRn receptor consists of the glycosylated heavy α-chain polypeptide, an MHC I class family member which associates with β2-microglobulin (β2m)^4,5^. IgG internalized by pinocytosis binds to FcRn at endosomal pH (5.5–6.0) and as a result, instead of being directed to the endolysosomal compartment for degradation, IgG:FcRn complexes are sorted into tubules originating from sorting endosomes and directed to return to the plasma membrane. Upon fusion with the plasma membrane, the intracellular fluid within the tubules is released and rapidly equilibrates with the extracellular pH 7.4 (refs. ^6–8^). At extracellular pH, the affinity of FcRn for the Fc domain is so low that antibodies are released back into circulation. This process occurs readily, despite the strong avidity effects of both the high local concentration of FcRn at the site of vesicle fusion, and the 2:1 stoichiometric binding of FcRn to IgG^9,10^. Biophysical studies have elucidated the molecular details of the IgG:FcRn interaction including the role of residues in the CH2–CH3 interface of the Fc domain in contact with FcRn, the pivotal role of His310 and His435 on pH-dependent binding, and the significance of protein dynamics^11–13^.

Engineering the human Fc domain to improve pharmacokinetic (PK) properties (manifest as an increased area under the plasma drug concentration–time curve (AUC), a lower clearance rate and a longer β-phase *T*_1/2_^14,15^) is of great interest for therapeutic purposes^3,16–20^. Better PK enable less frequent administration and lower dosing, which in turn translate into improved patient compliance and lower costs. Mutations of the Fc domain that enhance the affinity for FcRn at both the endosomal and physiological pH have been shown to result in greater antibody clearance. In contrast, Fc mutations that preferentially enhance FcRn affinity at pH 5.8 confer increased antibody half-life in circulation^16,18,20–23^. Fc domains containing the amino acid substitutions M428L/N434S (LS mutant), M252Y/S254T/T256E (YTE mutant), or H433K/N434F (KF mutant) confer 10- to 12-fold higher affinity for FcRn at pH 5.8, result in the greatest reported increase in antibody half-life (2- to 4-fold in circulation) in mice and in non-human primates, and are currently being evaluated in multiple clinical trials^16,24–26^. Notably, ravulizumab, a complement C5-inhibiting antibody containing the LS mutations, was FDA-approved for paroxysmal nocturnal hemoglobinuria and shown to have a serum half-life of ~49.7 days^27^, and MEDI8897, a respiratory syncytial virus (RSV)-neutralizing antibody containing the YTE mutations, was shown to have a serum half-life of ∼70 days, compared to approximately 20 days for wild-type (wt) IgG1, in infants in a phase 1b/2a study for prophylaxis against RSV infection^28^.

While further improvements in human antibody PK are highly desirable, extensive protein engineering campaigns focused on identifying mutations that confer even higher affinity for FcRn at pH 5.8 relative to the YTE or LS Fc variants have so far failed to yield amino acid combinations capable of imparting even longer circulation half-life in animal models^20,23,29–31^. Notably, YTE- and LS-Fc domains have significant binding to FcRn at pH 7.4 under high avidity conditions^11^. Residual binding at pH 7.4 may have a negative effect on plasma recycling and circulation persistence^32,33^. Another key consideration in the engineering of antibodies with ultra-long circulation half-life is that any mutation introduced into the Fc domain should not negatively impact other antibody characteristics such as effector functions or manufacturability^34^. For example, the YTE, LS, and KF mutations reduce FcγR binding and effector functions to various degrees (depending on the mutant antibody and patient FcγR allotype), and the YTE mutations have been reported to also reduce complement-dependent cytotoxicity (CDC)^18,35^.

Antibody PK are routinely evaluated in transgenic (Tg) mice overexpressing human FcRn (hFcRn) from non-native promoters^36,37^. However, the currently available hFcRn Tg mouse models do not recapitulate many of the mechanisms that affect antibody clearance. Key limitations of the existing mouse models used for antibody PK studies arise from: (i) non-physiological levels of transgene hFcRn expression relative to mouse FcRn in wild-type animals^38^; (ii) expression of mouse β2m instead of human β2m (hβ2m) in many of the currently used models^36,37,39^; (iii) absence of endogenously produced human IgG to compete for hFcRn binding^40^; and (iv) no ability to account for the effect of binding to human FcγRs in clearance^41,42^. To address these limitations, as part of this work we constructed new knock-in mouse models that express hFcRn, hβ2m, and hFcγRs; and, in addition, produce endogenously chimeric mouse–human IgG1, composed of mouse variable regions and human constant regions for the heavy and light chain (hIgG1,κ).

Here, we report the engineering of a human IgG Fc domain which, by virtue of having moderately higher affinity for FcRn at pH 5.8 but no detectable binding at pH 7.4 under high avidity conditions, confers improved antibody PK properties compared to the clinical stage YTE and LS variants, both in the commonly used hFcRn^Tg^ mouse model (hemizygotic 276) as well as in our new knock-in model (hFcRn-hβ2m-hFcγRs-hIgG1,κ mice). Importantly, we show that antibodies utilizing our engineered ultra-long half-life Fc domain display the full range of effector functions as the wt Fc domain while exhibiting far favorable biophysical properties for clinical development.

## Results

### Pharmacokinetics properties of an engineered human Fc variant

**Fig. 1:**
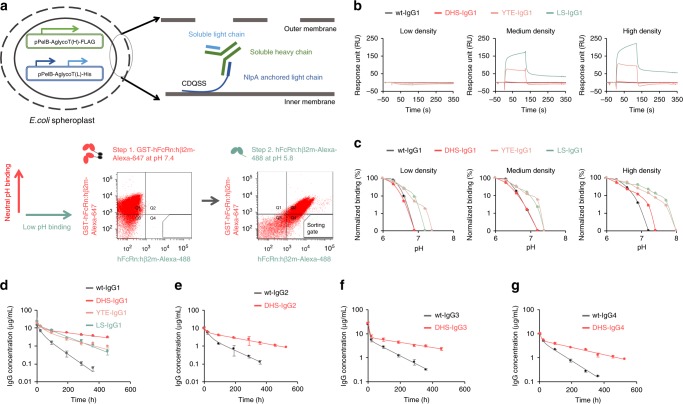
Engineering a human Fc domain with optimized pH-dependent FcRn binding for ultra-long circulation persistence. **a** Screening strategy for the isolation of Fc mutations that confer favorable pH-dependent FcRn-binding using *E. coli* display. **b**, **c** SPR binding of IgG mutants (800 nM) to hFcRn:hβ2m immobilized at low, medium, or high density (500, 2000, and 4000 RU, respectively) either **b** at pH 7.4 or **c** as a function of pH. Normalized binding intensity was calculated as the pH-dependent RU over the RUmax at pH 6.0, for antibodies at 800 nM. Error bars: standard deviation from three independent experiments. **d**–**g** Serum antibody concentration of DHS formatted IgG1 (**d**), IgG2 (**e**), IgG3 (**f**), and IgG4 (**g**) antibodies in hemizygotic Tg276 hFcRn transgenic mice as a function of time after administration. Each antibody variant (2 mg/kg) was administered intravenously to hemizygous Tg276 mice (*n* = 11 for IgG1 and *n* = 5 for IgG2, IgG3, and IgG4). Antibody concentrations were determined by ELISA.. Data are presented as mean ± standard deviation

We developed a strategy that capitalizes on *Escherichia coli* display of large combinatorial IgG libraries (Anchored Periplasmic Expression (APEx) technology^43–46^) for the isolation of clones expressing human IgG1 with mutated Fc domains that bind selectively to the human FcRn/human β2m complex (hFcRn:hβ2m) at pH 5.8 but not at pH 7.4 (Fig. 1a). Select regions of the human IgG1 C_H_2–C_H_3 hinge were combinatorially mutagenized (Supplementary Fig. 1a–c and Supplementary Table 1) to create a library of >10^8^ transformants. Briefly, *E. coli* spheroplasts expressing mutated human IgG1 (Trastuzumab) anchored on the external leaflet of the inner membrane were first screened by FACS for binding to Alexa488-labeled hFcRn:hβ2m. Three rounds of FACS with hFcRn:hβ2m at pH 5.8 were performed to enrich antibodies with Fc domains with higher binding affinity at pH 5.8. The pool of clones enriched for enhanced binding at endosomal pH was then subjected to a competitive, two-step labeling process to eliminate variants that have detectable binding at pH 7.4 to high avidity, dimeric, GST-hFcRn:hβ2m. For this purpose, spheroplasted cells were labeled with an excess of Alexa647-labeled GST-hFcRn:hβ2m (red) at pH 7.4 and then spheroplasts were washed with pH 7.4 phosphate-buffered saline (PBS). The spheroplasts were subsequently labeled with monomeric hFcRn:hβ2m-Alexa488 (green) at pH 5.8, and clones with high green fluorescence and low red fluorescence (i.e. absence of residual Alexa647-labeled GST-hFcRn:hβ2m from the first labeling step) were isolated and characterized (Fig. 1a). Four clones expressing different Fc variants from the last round of screening were isolated, individually confirmed to display pH-dependent binding by FACS, and were all found to share three amino acid substitutions: V264E, L309D, and Q311H (EDH) (Supplementary Fig. 1d).

The four isolated antibody variants were produced in HEK293 cells. One variant, EDHY-IgG1 (V264E, L309D, Q311H, and N434Y), had a ~20-fold better affinity (*K*_D,pH5.8_ = 28 nM) for hFcRn at pH 5.8 compared to wt IgG1 (*K*_D,pH5.8_ = 550 nM) but also had residual binding at pH 7.4. A second variant, EDHS-IgG1 (V264E, L309D, Q311H, and N434S), had a ~6-fold increased affinity at 5.8 (*K*_D,pH5.8_ = 93 nM) but no apparent binding at pH 7.4 (Supplementary Fig. 1e–g). Very extensive mutagenesis efforts aimed at identifying mutations conferring even better *K*_D,pH 5.8_ were unsuccessful, as all of the mutants isolated based on higher affinity at pH 5.8 had detectable binding at pH 7.4 based on FACS analysis.

Antibodies with the EDHS mutations in the Fc had drastically reduced binding to effector hFcγRs (Supplementary Fig. 1h), which was found to be due to the V264E mutation^47^. Since a V264E substitution has a minimal effect on hFcRn binding at pH 5.8 (ref. ^48^), it was reverted back to a valine to yield the L309D/Q311H/N434S (DHS) Fc domain, resulting in complete recovery of hFcγR binding (Supplementary Fig. 1h). Moreover, these three amino acids substitutions did not affect the N-glycosylation pattern of the Fc domain which was comparable to that of wt IgG1 antibodies (Supplementary Fig. 2).

We performed a detailed analysis of the hFcRn:hβ2m-binding kinetics of DHS antibodies as a function of pH using SPR analysis with immobilized hFcRn:hβ2m at low, medium, or high density^11^ (500, 2000, or 4000 RUs, Fig. 1b–d and Supplementary Fig. 3). Consistent with earlier reports^49^, YTE- and LS-IgG1 showed moderate and significant binding, at pH 7.4, respectively when SPR analysis was performed with medium or high hFcRn:hβ2m densities (Fig. 1b, c, Table 1). In contrast, we could detect no binding of DHS- or wt-IgG1 to hFcRn:hβ2m at physiological conditions at even the highest density tested (Fig. 1b).

**Table 1:** *K*_D_ values for binding of DHS, YTE, LS variants or wt IgG1 to hFcRn:hβ2m dimer at pH 5.8 and 7.4

*K*_D_ (nM)	pH 5.8	pH 7.4		
		Low density	Medium density	High density
wt-IgG1	550 ± 50	ND	ND	ND
DHS-IgG1	110 ± 20	ND	ND	ND
YTE-IgG1	23 ± 1	ND	4730 ± 480	4290 ± 310
LS-IgG1	55 ± 3	ND	946 ± 170	567 ± 152

All IgG variants analyzed with hFcRn:hβ2m dimer and their kinetic values were analyzed by the equivalent binding model. Data are presented as mean ± standard deviation from triplicate experiments

The PK of Trastuzumab, which does not bind any mouse targets, formatted with DHS-, YTE-, LS-, or wt-IgG1 Fc domains were first evaluated in hemizygous hFcRn^Tg^ mice (hemizygotic Tg276), a widely used Tg mouse model for comparing the circulation persistence of human antibodies^37^. Following administration of a single intravenous dose (i.v. 2 mg/kg) by tail vein injection, the β-phase *T*_1/2_ (β-phase half-life) of DHS was 2.0- and 3.1-fold higher compared to YTE and LS variants (290.9 ± 25.6 h compared to 148.4 ± 36.8 and 92.9 ± 6.1 h, respectively, data are presented as mean ± standard deviation). Total drug exposure over time for DHS-IgG1 (AUC_inf_) was 1.6-, 1.9-, and 5.3-fold greater relative to YTE-, LS-, and wt-IgG1 variants, respectively (Fig. 1d, Table 2). Antibody clearance followed biphasic kinetics, with a clearance rate of 0.11 ± 0.01 mL/day/kg for DHS-IgG1, approximately 6-fold lower than wt IgG1, and 1.9- and 2.2-fold lower than YTE- and LS-IgG1. Introduction of the DHS mutations into the Fc domain of IgG2, IgG3 (G3m16 allotype with H435 residue), or IgG4 subclasses also resulted in no detectable interaction with FcRn at pH 7.4, and likewise, conferred significantly improved PK for all the other three IgG subclasses (Fig. 1e–g, Supplementary Fig. 4, Supplementary Table 2). Collectively, our data: (a) reveal that the DHS Fc domain imparts improved antibody PK properties in the hemizygous Tg276 hFcRn^Tg^ model relative to the best in class clinical stage, half-life extension variants YTE and LS and (b) support the hypothesis^32,33^ that residual binding to FcRn at physiological pH has a major detrimental effect on antibody circulation persistence.

**Table 2:** Pharmacokinetic parameters for hemizygotic Tg276 hFcRn transgenic mice

	AUC_inf._ (μg days/mL)	Clearance (mL/day)	β-phase *T*_1/2_ (h)	*V*_ss_ (mL/kg)
wt-IgG1	30.0 ± 1.6	0.66 ± 0.17	49.6 ± 13.4	1.50 ± 0.15
DHS-IgG1	157.9 ± 3.4	0.11 ± 0.01	290.9 ± 25.6	0.59 ± 0.11
YTE-IgG1	96.9 ± 3.3	0.24 ± 0.03	148.4 ± 36.8	0.72 ± 0.14
LS-IgG1	84.4 ± 2.0	0.21 ± 0.03	92.9 ± 6.1	0.60 ± 0.06

Data are presented as mean ± standard deviation (*n* = 11)

### Pharmacokinetic studies in novel knock-in mouse models

**Fig. 2:**
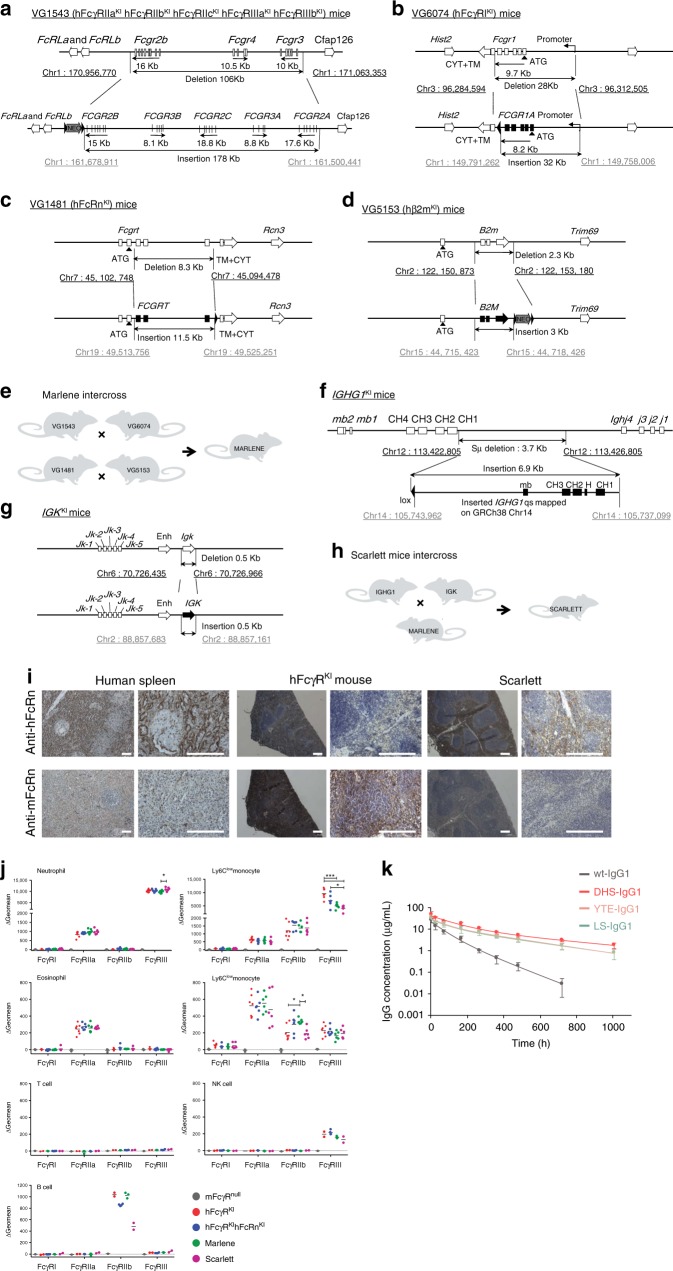
IgG pharmacokinetics in new knock-in mouse models. **a**–**h** Generation of knock-in mice for human FcγRs, FcRn, β2m, IgG1 heavy chain and kappa light chain constant regions: **a** humanization of the mouse low-affinity receptor locus; **b** humanization of the ectodomains of mouse FcγRI; **c** humanization of the ectodomains of mouse FcRn; **d** humanization of the mouse β2m gene (B2m); **e** breeding strategy to generate Marlene mice; **f** replacement of the switch μ region of the mouse heavy chain locus by the constant region of human IgG1 heavy chain (IGHG1); **g** humanization of the kappa light chain gene (IGK); **h** breeding strategy to generate Scarlett mice; **a**–**d**, **f**–**g** representations are not drawn to scale. Coordinates are based on mouse (GRCm38.p4) and human (GRCh38.p7) genomic assemblies; mouse genes are in empty rectangles, genomic coordinates are in black; human genes are in solid rectangles, genomic coordinates are in gray, black triangles represent LoxP sites. mb, exon encoding the transmembrane and intracytoplasmic domains; H, exon encoding the hinge domain; Neo, selection cassette. Mouse silhouette was created by co-author P.B. **i** Immunohistochemical localization of hFcRn or mFcRn in tissue sections of human spleen, and spleen sections from of hFcγR^KI^ mice (VG1543 × VG6074) and Scarlett mice. Scale bar = 200 μm. **j** hFcγR expression on different cell populations from the spleen of FcγR^null^ (negative control), hFcγR^KI^ (positive control), hFcRn^KI^ hFcγR^KI^, Marlene and Scarlett mice. *P* values by one-way Anova with Tukey’s multiple comparison tests, *P ≤ 0.05 and ***P ≤ 0.001. **k** Change in serum IgG concentration following tail vein administration of 2 mg/kg of antibody in Scarlett (hFcRn^KI^ hβ2m^KI^ hFcγR^KI^ hIgG1, κ^KI^) mice (*n* = 6). Data are presented as mean ± standard deviation

To more accurately evaluate antibody PK we constructed knock-in (KI) mouse models that account for multiple processes known to affect human IgG clearance and homeostasis (Fig. 2a–e). In these KI mouse strains: (i) the hFcRn:hβ2m complex is expressed in a manner that faithfully reflects mouse FcRn levels, (ii) the human effector FcγR family is expressed to account for FcγR-dependent clearance, and finally, (iii) human IgG1, which, unlike murine IgG, competes with administered therapeutic antibodies for hFcRn binding^40^, is produced endogenously. We developed a mouse in which the murine FcγR genes had been exchanged with the corresponding human genes, including introns and regulatory elements for *FCGR1A* and for *FCGR2A, FCGR2B, FCGR2C, FCGR3A*, and *FCGR3B* genes (hFcγR^KI^ mouse, intercross of VG6074 and VG1543 mice^50^; Fig. 2a, b)^51^. The *Fcgrt* (mouse FcRn gene) was replaced with *FCGRT* (human FcRn gene, VG1481; Fig. 2c) and the *B2m* (mouse β2m gene) was replaced with *B2M* (human β2m gene, VG5153; Fig. 2d). The hFcRn^KI^ hβ2m^KI^ hFcγR^KI^ mice (designated as Marlene mice) were bred by crossing the four transgenes as shown in Fig. 2e. Finally, to account for the fact that endogenous mouse IgG does not compete with human IgG for binding to human FcRn^52^, the heavy-chain constant region of human IgG1 (*IGHG1*) was knocked-in to replace the switch μ region of the mouse heavy-chain locus, and the human kappa light-chain constant region (*IGK*) was knocked-in to replace the mouse kappa light-chain region (kappa light chains are used in 95% of circulating antibodies in mice), thereby generating mice that exclusively produce human IgG1 heavy chains, predominantly associated to human kappa light chains (Fig. 2f, g). The hFcRn^KI^ hβ2m^KI^ hFcγR^KI^ hIgG1,κ^KI^ mice (designated as Scarlett mice) were bred by crossing the Marlene and the hIgG1,κ^KI^ mice (Fig. 2h). hFcRn expression was confirmed by immune-histochemistry and RT-PCR of spleens of Scarlett mice (Fig. 2i, Supplementary Fig. 5). Cell type-specific expression of hFcγRs in Marlene and Scarlett is reported in Fig. 2j and Supplementary Fig. 6 is similar to that of humans, and consistent with our earlier reported expression pattern for hFcγR^KI^ mice^50^. Scarlett mice displayed 440 ± 120 μg/mL of human IgG1 in serum and undetectable levels of mouse IgG. The mouse IgG serum concentration in control C57BL/6J mice was 605 ± 147 μg/mL, in the same order of magnitude as human IgG1 in Scarlett mice.

We observed very comparable β-phase elimination half-life in the KI animals that produce endogenous human antibodies (Scarlett) for three different approved IgG1 antibodies (Trastuzumab, Rituximab, or Omalizumab) after a single intravenous dose (i.v. 2 mg/kg) by tail vein injection. Specifically, for Trastuzumab we compared the PK between the Scarlett mice and animals that do not have circulating human IgG1 (Marlene mice; Supplementary Fig. 7). The β-phase elimination half-life was 40% lower in the Scarlett mice underscoring how competition for FcRn binding by circulating human IgG1 impacts the PK of administered antibodies in hFcRn^Tg^ mice.

Consistent with the trends observed in the hemizygous Tg276 hFcRn^Tg^ mouse model, the data in Fig. 2k and Table 3 reveal that in Scarlett mice -DHS antibodies have ≥50% longer β-phase half-life (*P* ≤ 0.0001 to LS- or YTE-IgG1), a proportional increase in AUC_inf_ (*P* ≤ 0.0001 to LS- or YTE-IgG1), and a proportional decrease in clearance rate (*P* ≤ 0.0001 to LS- or YTE-IgG1) relative to the YTE and LS variants. Compared to wt IgG1, the DHS mutations resulted in fourfold longer β-phase half-life and fivefold greater AUC_inf_.

**Table 3:** Pharmacokinetic parameters for Scarlett (hFcRn^KI^ hβ2m^KI^ hFcγR^KI^ hIgG1, κ^KI^) mice

	*C*_max_ (mg/mL)	AUC_inf_ (μg days/mL)	Clearance (mL/day)	β-phase *T*_1/2_ (h)	*V*_ss_ (mL/kg)
wt-IgG1	29.9 ± 9.2	74.8 ± 7.35	0.27 ± 0.03	92.1 ± 12.3	0.65 ± 0.04
DHS-IgG1	45.6 ± 18.4	356.8 ± 16.7	0.056 ± 0.003	381.0 ± 85.0	0.25 ± 0.01
YTE-IgG1	35.5 ± 11.6	238.1 ± 15.9	0.084 ± 0.006	236.8 ± 20.8	0.33 ± 0.02
LS-IgG1	40.2 ± 12.6	223.0 ± 24.1	0.090 ± 0.009	255.0 ± 31.8	0.36 ± 0.02

Data are presented as mean ± standard deviation (*n* = 6)

### Effector functions and biophysical properties of DHS-Fc

**Fig. 3:**
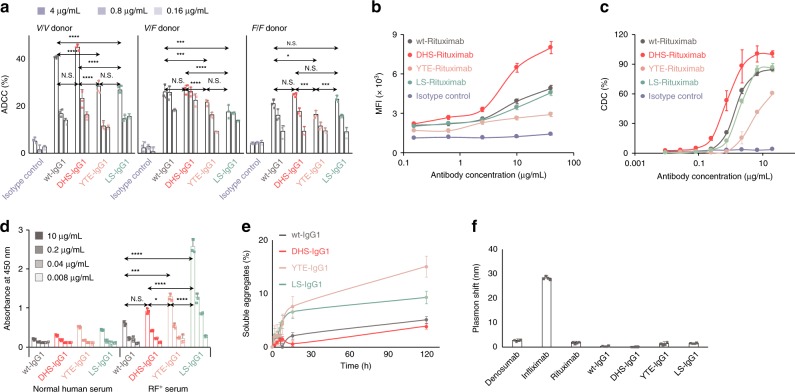
Features of DHS Fc variants relevant to therapeutic antibody development. **a** ADCC assay of SK-BR-3 with PBMCs from FcγRIIIa *V/V*, *V/F*, or *F/F* donors. **b** C1q deposition on CD20^+^ Raji cells revealed by flow cytometry. **c** CDC assay of Rituximab-Fc variants with Raji cells as a function of antibody concentration. **d** Binding to rheumatoid factor (RF) measured by ELISA. *P* values by two-way ANOVA test, NS *P* ≥ 0.05, *P ≤ 0.05, **P ≤ 0.01, ***P ≤ 0.001, and ****P ≤ 0.0001. **e** Extent of antibody aggregation following thermal stress (50 °C incubation) of Trastuzumab variants, measured by size exclusion chromatography (SEC). **f** Antibody self-association properties measured by affinity-capture self-interaction nanoparticle spectroscopy (AC-SINS) assay. Data are from one experiment representative of three experiments using three individual donors (**a**–**d**). Errors in all plots and tables represent standard deviations from triplicate experiments

Mutations that favorably impact antibody serum half-life can adversely affect binding to effector hFcγRs, in turn impairing the clearance of target cells by ADCC or ADCP^35^. Consistent with earlier reports, SPR measurements showed that the YTE mutations reduce binding to the low-affinity FcγRs (i.e. hFcγRIIa, hFcγRIIb, hFcγRIIIa) by more than threefold. The LS substitutions have a more modest effect on hFcγR binding, resulting in a twofold reduction in affinity for hFcγRI and hFcγRIIa_H131_, and threefold reduction for hFcγRIIIa_V158_. In contrast, the affinities of the DHS-IgG1 for hFcγRs were statistically indistinguishable (or slightly improved in the case of hFcγRIIIa_F158_) compared to those of wt-IgG1 (Table 4, Supplementary Fig. 8a). Class-switched IgG2, IgG3, or IgG4 variants of Trastuzumab bearing DHS mutations also showed statistically indistinguishable binding to hFcγRs relative to their wt counterparts (Supplementary Fig. 8b). Not surprisingly, the in vitro ADCC activity of DHS-Trastuzumab IgG1 was identical to that observed with wt Trastuzumab in hFcγRIIIa-*V/V* and -*V/F* donors, but greater than that of YTE-Trastuzumab IgG1 or LS-Trastuzumab IgG1 (Fig. 3a). On the other hand, as expected^35^, with homozygotic hFcγRIIIa-*F/F* donors, DHS-Trastuzumab IgG1 showed equivalent ADCC activities with wt Trastuzumab or LS-Trastuzumab IgG1 (Fig. 3a).

**Table 4:** *K*_D_ values for human FcγRs determined by SPR

*K*_D_ (μM)	FcγRI (×10^−3^)	FcγRIIa_H131_	FcγRIIa_R131_	FcγRIIb	FcγRIIIa_F158_	FcγRIIIa_V158_
wt-IgG1	0.21 ± 0.02	0.22 ± 0.06	1.16 ± 0.25	2.64 ± 0.39	1.17 ± 0.23	0.17 ± 0.04
DHS-IgG1	0.28 ± 0.02	0.27 ± 0.04	0.95 ± 0.02	2.91 ± 0.54	0.48 ± 0.07	0.18 ± 0.03
YTE-IgG	0.29 ± 0.02	0.62 ± 0.18	3.20 ± 0.26	6.24 ± 0.10	4.41 ± 0.93	0.82 ± 0.11
LS-IgG1	0.58 ± 0.12	0.41 ± 0.30	1.21 ± 0.02	2.25 ± 0.03	0.64 ± 0.09	0.52 ± 0.09

All IgG variants analyzed with monomeric Fc receptors and their kinetic values were analyzed by the equivalent binding model. Data are presented as mean ± standard deviation from triplicate experiments

Next, we examined the ability of anti-CD20 (Rituximab) antibody variants to activate the classical complement pathway via the recruitment of C1q. Target cells (CD20^+^ Raji) opsonized with DHS-Rituximab IgG1 antibodies had a higher level of cell surface-deposed C1q relative to wt-, LS-, or YTE-Rituximab antibodies. DHS-Rituximab displayed twofold more potent CDC (0.7 ± 0.1 μg/mL) than wt- (1.5 ± 0.1 μg/mL) or LS-Rituximab (1.9 ± 0.1 μg/mL), and 10-fold more potent CDC than YTE-Rituximab, the latter which has already been reported to display significantly lower CDC activity than wt-Rituximab^18,48^ (Fig. 3b, c).

Rheumatoid factor (RF) refers to auto-antibodies that bind to the Fc domain of immunoglobulins. The binding of RF to therapeutic antibodies is associated with faster clearance and increased antigen presentation and anti-drug antibodies^53–56^. We measured the RF binding of the wt-, DHS-, YTE-, and LS-IgG1 by ELISA using polyclonal sera from donors with high RF titers. DHS-IgG1 showed binding to RF sera that was indistinguishable from that of wt-IgG1 and significantly lower than that observed with YTE-IgG1 and especially with LS-IgG1 (Fig. 3d).

Size exclusion chromatography (SEC) showed that for all four IgG subclasses, introduction of the DHS mutations did not affect protein solubility nor did it lead to soluble aggregate formation (Supplementary Fig. 9a). The thermodynamic stability of therapeutic antibodies is one of the most important biophysical properties for clinical development^34,57,58^. The melting temperature (*T*_m_) was comparable for the wt, DHS, and LS^35^ Fc variants (Table 5) but was more than 6 °C lower for the YTE variant (Table 5)^34^. The susceptibility of the antibody variants to aggregation at 70 °C was very high for YTE-IgG1, leading to visible precipitates within minutes, intermediate for LS-IgG1, and minimal for wt- and DHS-IgG1 (Supplementary Fig. 9b–e). Similarly, the YTE- and LS-IgG1 formed higher levels of soluble aggregates upon prolonged incubation at 50 °C (Fig. 3e). Finally, the self-association propensity of the various engineered Fc antibodies at room temperature was determined by affinity-capture self-interaction nanoparticle spectroscopy (AC-SINS), a widely used assay for antibody developability^58–60^. Earlier AC-SINS analyses using 137 FDA-approved antibody drugs found that AC-SINS plasmon shifts >11.8 nm correspond to antibodies with high levels of self-association and which represents a development risk^58^. For DHS-IgG1 the AC-SINS plasmon shift was only 0.1 ± 0.5 nm; of note, YTE- and LS-antibodies also showed low plasmon shift in this assay albeit higher than that of DHS-IgG1 (Fig. 3f).

**Table 5:** Tm measurement by differential scanning fluorimetry (DSF)

	*T*_m_ (°C)
wt-Fc	69.0 ± 0.2
DHS-Fc	70.2 ± 0.2
YTE-Fc	62.4 ± 0.2
LS-Fc	67.9 ± 0.2

Data are presented as mean ± standard deviation (*n* = 3)

Lee et al. report an engineered IgG1 Fc domain that behaves like an hFcRn binding pH toggle switch. The authors show that this new half-life extension Fc domain confers improved pharmacokinetics in new humanized knock-in mouse strains that recapitulate the key processes for antibody persistence in circulation

## Discussion

An ideal Fc domain for therapeutic applications must confer optimal pH-dependent binding to hFcRn to achieve very long circulation half-life, maintain intact effector functions, and display favorable biophysical properties suitable for clinical development^57,58^. To enable maximal antibody circulation half-life, an Fc domain should resemble a pH toggle switch with a low FcRn *K*_D,pH5.8_ and no detectable binding activity at pH 7.4. However, despite extensive efforts, engineering Fc domains with a lower FcRn *K*_D,pH 5.8_ and no detectable binding at pH 7.4 under high avidity conditions had proven elusive^11,20^. Here, we used a two-step high-throughput screening strategy to isolate the DHS Fc variant, which has a fivefold lower *K*_D,pH5.8_ for FcRn relative to wt-IgG1 Fc and no detectable binding at pH 7.4. Unlike antibodies containing the YTE or LS half-life extension mutations, the DHS Fc domain does not bind to high densities of immobilized hFcRn at pH 7.4 (Fig. 1b, c, Table 1). We found that, by virtue of the lack of detectable binding at pH 7.4 and despite having a lower affinity for FcRn at endosomal pH relative to the LS or YTE variants, DHS antibodies exhibit even more favorable pharmacokinetics in multiple mouse models. Specifically, as shown in Fig. 1d and Table 2, the DHS Fc domain confers significantly improved antibody PK properties relative to the YTE and LS variants in hemizygous Tg276 mice, a widely used model for antibody PK studies^37^. In the Tg276 model, hFcRn is transcribed from the CAG promoter, resulting in ubiquitous high-level gene expression^36^. A second transgenic mouse line, Tg32, in which hFcRn is under the control of the human hFcRn promoter, is also commonly used for antibody PK studies^46^; however, in this model the transgene is inserted in close proximity to a mouse enhancer region which in turn impacts transcription and tissue-specific expression. Because neither model fully recapitulates mouse endogenous FcRn expression^38^, we constructed new models in which hFcRn and hβ2m are expressed from the respective mouse promoters. The binding of antibodies to FcγRs, and in particular to FcγRs expressed by liver-resident cells, is known to affect antibody clearance^41,42^. However, since human antibodies bind to mouse FcγRs with very different affinities than those of their human counterparts^61,62^, the effect of FcγR-mediated clearance of human IgG cannot be properly accounted for in animals expressing murine IgG receptors. To address this shortcoming, we further engineered hFcRn^KI^ hβ2m^KI^ mice to also express all the human FcγR family proteins (Marlene mice). Yet another factor that plays an important role for therapeutic antibody PK is the competition for hFcRn binding by endogenous, circulating human IgG. Because mouse IgG does not bind to hFcRn^40^ in some earlier antibody PK studies in Tg276 or Tg32 hFcRn^Tg^ mice intravenous co-administration of IgG (IVIG) was attempted as a surrogate for endogenous IgG competition. However, the effect of IVIG was reported to be dependent on the mouse model and was more pronounced in animals expressing lower hFcRn levels^40,63^. To overcome these problems, we further engineered the Marlene mice to express chimeric mouse-human IgG1 containing the constant regions of human IgG1 heavy chain and human kappa light chain. The resulting Scarlett mouse strain displayed circulating human IgG1 concentrations comparable to those of mouse IgG1 in C57BL/6J mice. We note however that even though the Scarlett model produce levels of human IgG1 comparable to what is physiologically relevant for mouse IgG1, it may nonetheless somewhat underestimate the impact of competing IgG1 in humans due to species differences since humans produce higher levels of this antibody isotype. Beyond their utility in comparing the PK of different half-life extension variants as shown here, these new knock-in models are likely to prove particularly useful for preclinical studies for the understanding of antibody effector functions in mice that express physiologically relevant levels of hFcγRs and additionally express competing endogenous IgG1.

Consistent with the improved PK properties of antibodies with the DHS mutations compared to antibodies with the clinical-stage YTE and LS variants in Tg276 hemizygous mice in Fig. 1d and Table 2, the DHS amino acid substitutions resulted in ≥50% increase of β-phase *T*_1/2_ and AUC_inf_ in the Scarlett model. The relative improvement in the PK parameters of DHS antibodies compared to the YTE or LS variants in the Scarlett model was lower than the effect (twofold) observed in Tg276 and may be due to competition by IgG, the impact of effector FcγR clearance mechanisms and possibly, mouse strain background differences. If, as expected, the PK behavior of antibodies in Scarlett mice more closely mimics that in humans, the observed 50% greater β-phase half-life and drug exposure afforded by the DHS mutations should result in significant therapeutic benefit. For example, a 50% improvement over YTE antibodies^28^ could be expected to translate into a circulation half-life in humans of well over 100 days.

To quantitatively explore the significance of endosomal retention (determined by *K*_D_ at pH 5.8) versus surface recapture (determined by the *K*_D_ at pH 7.4) we constructed a simple phenomenological PK model (Supplementary Fig. 10). This model is sufficient to explain the relative PK properties of each Fc domain. Sensitivity analysis indicates that surface recapture is the dominant parameter limiting circulation persistence of YTE- and LS-IgG1 mutants. Specifically, in the hemizygotic Tg276 mice as well as in the Scarlett mice, the consequence of residual affinity of these Fc domains at the extracellular pH is roughly 50% or 20% recapture, respectively, of antibody on the cell surface during endosomal recycling. Collectively, both experimental PK comparison and the quantitative modeling of antibodies with distinct hFcRn-binding properties at endosomal and physiological pH highlight the negative effect of hFc:hFcRn binding at pH 7.4 on antibody circulation persistence.

Fc-mediated effector functions (ADCC and CDC), developability (biophysical properties including thermodynamic stability), and drug bioavailability (serum half-life/AUC_inf_) are of critical importance for clinical application of therapeutic antibodies^58,64^. We demonstrate herein that the DHS Fc variant has (i) intact Fc-mediated effector functions, (ii) a similar Tm, low aggregation propensity, no evidence for self-association and low binding to RF, similar to wt IgG1 antibodies, and (iii) ultra-long circulation persistence in highly relevant new humanized mouse models.

## Methods

### Cells and reagents

Adenocarcinoma SK-BR3 cell line (ATCC^®^ HTB-30™) and Raji cell line (ATCC® CCL-86™) were obtained from American Type Culture Collection. SK-BR3 was cultured in complete Dulbecco's modified Eagle's medium (DMEM) with 10% fetal bovine serum (FBS) and Raji was cultured in complete RPMI 1640 with 10% FBS. Human PBMCs were purified from anonymous healthy volunteers using histopaque density gradient centrifugation (Sigma-Aldrich). Human C1q (CompTech) and hFcRn:hβ2m (Novus Biologicals) were purchased. All primers were synthesized by Integrated DNA Technologies.

### Preparation of recombinant proteins

Human Fc receptors, including hFcγRI, hFcγRIIa, hFcγRIIb, hFcγRIIIa, and hFcRn:hβ2m, were cloned in-frame into the mammalian expression vector pcDNA3.4 using Gibson Assembly cloning (NEB)^44^ and were produced by transient transfection of Expi293 cells (Invitrogen) according to the manufacturer’s instruction^43,44,65^. His-tagged Fc receptor proteins were purified with Ni-NTA (GE Healthcare) affinity columns and GST-tagged Fc receptor proteins were purified with Glutathione Sepharose (GE Healthcare) affinity columns according to the manufacturer’s instructions. In order to remove lipopolysaccharide (LPS) and non-specifically bound protein, the Fc receptor-bound resins were washed with 50 mL of PBS containing 0.1% Triton®X-114 (Sigma-Aldrich) and 50 mL of PBS. His-tagged Fc receptor proteins were eluted with PBS containing 250 mM imidazole and GST-tagged Fc receptor proteins were eluted with PBS containing 10 mM reduced l-glutathione. The buffer in the Fc receptor protein containing eluent fraction was exchanged with PBS by Amicon Ultra-30 centrifugal spin columns (Milipore).

Similarly, isolated Fc candidates were cloned in-frame into the mammalian expression vector pcDNA3.4-IgH-Trastuzumumab using Gibson Assembly cloning (NEB)^66^ and were produced in Expi293 cells (Invitrogen) grown for 5 days at 37 °C with 8% CO_2._ All IgG variants proteins purified by Protein A high capacity agarose resin (Thermo Scientific) according to the manufacturer’s instructions. Purity of antibody variants was confirmed by SDS-PAGE and size exclusion chromatography and was over 95%.

### Library construction and screening

Amino acid residues D249-R255, V308-L314, and E430-Q438 in the Fc that are within 7 Å distance from FcRn in the crystal structure of the human Fc:FcRn complex (PDB ID: 4N0U)^13^ were selected for mutagenesis using the aa substitution scheme shown and three separate libraries were constructed using primers with degenerate codons (Supplementary Table 1). The heavy chain of trastuzumab IgG1 was used as the template. The amplified PCR fragments were ligated into pPelB-AglycoT(H)-FLAG^43^ with *Sfi*I restriction sites for the histidine scanning library. The resulting plasmids were transformed into *E. coli* Jude-1(F′ [Tn*10(*Tet^r^*)* proAB^+^ *lacI*^q^ Δ(*lacZ*)M15] *mcrA* Δ(*mrr*-*hsdRMS*-*mcrBC*) 80d*lac*ZΔM15 Δ*lacX74 deoR recA1 araD139* Δ(*ara leu*)7697 *galU galK rpsL endA1 nupG*) harboring pBAD-AglycoT(L)-His^43,67^ to display both heavy and light chain of aglycosylated IgG1 in periplasm anchored on the inner membrane via the NlpA signal peptide (APEx display)^45^. Following electroporation 1 × 10^8^ transformants were obtained. The transformants were spheroplasted after overnight induction with 1 mM of IPTG and 0.2% of l-arabinose, followed by screening with 10 nM of hFcRn:hβ2m, conjugated with fluorescent Alexa488 (hFcRn:hβ2m Alexa488) as a target probe, at pH 5.8 in PBS. Screening was performed using a FACSAria™ (BD Biosciences). In each round, the top 3% of the population showing the highest fluorescence was collected and the spheroplasts were re-sorted immediately to remove false positives. The transformants with the plasmids of sorted spheroplasts were prepared. After four rounds, in order to achieve pH-selective binding activity, the library was first labeled with 5 nM of GST-hFcRn:hβ2m-Alexa647 at neutral pH, then incubated with 10 nM of hFcRn:hβ2m-Alexa488 at pH 5.8 (Fig. 1a). After the fifth round, single Fc variants were analyzed with pH-dependent-binding activities by flow cytometry and the four candidates were isolated.

### LC-ESI-MS/MS analysis of Fc variants

Fc proteins were analyzed by The University of Texas at Austin Proteomics Facility using LC-MS on Thermo Orbitrap Fusion Tribrid mass spectrometer with the FT detector. A fast gradient of 0.1% formic acid/water and 0.1% formic acid/acetonitrile over 10 min was used to elute the intact proteins from an OPTI-TRAP^TM^ protein microtrap (Optimize Technologies). The Orbitrap Fusion was operated in Intact Protein Mode at 15,000 resolution from 400 to 2000*m*/*z*. The data were deconvoluted using Thermo Protein Deconvolution software.

### Size exclusion chromatography

SEC analyses for the purified IgG proteins were performed on an ÄKTA Pure (GE Healthcare) liquid chromatography system using a Superdex 200 10/300GC, (GE Healthcare), with a mobile phase of pH 7.4 PBS at a flow rate of 0.75 mL/min. Chromatograms were obtained by monitoring the absorbance at 280 nm. The injection amount was 100 μg of proteins in a volume of 200 μL. For thermal stress test, each antibody protein (1 mg/mL) was incubated at 70 °C for 10, 20, 40, and 60 min and then analyzed by SEC using the above described method.

### Differential scanning fluorimetry

Differential scanning fluorimetry (DSF) was performed with SYPRO Orange dye (Catalog #S-6650; Life Technologies) to determine the thermal stability by monitoring the protein unfolding of the purified Fc domains. For each sample, 15 μL of Fc domains varying from 0.08 to 20 μM were mixed with 5 μL of dye, diluted from 5000× to 20× for use. Each experiment included a control without proteins containing 100 mM HEPES buffer at pH 7. The DSF assays were performed on an Applied Biosystems ViiA 7 Real-Time PCR System using a temperature gradient from 25 to 99 °C at a temperature ramp rate of 0.03 °C/s. The fluorescence emissions and melting temperature measurements were analyzed using ViiA 7 Software from Life Technologies.

### AC-SINS

Goat anti-human Fc fragment specific (Jackson ImmunoResearch, 109-005-008) was buffer exchanged into 20 mM potassium acetate (pH 4.3) twice using Zeba desalting columns (Thermo Fisher Scientific, PI-89882). The goat anti-human Fc concentration was evaluated using UV absorbance value at 280 nm (extinction coefficient of 1.26 mL/mg cm). The polyclonal antibody was diluted to 0.4 mg/mL in the acetate buffer. Equal volumes of goat anti-human Fc antibody and gold nanoparticles (Ted Pella, 15705-1) were incubated at room temperature (1 h). The antibody-gold conjugates (3 mL) were loaded onto 0.22 μm filters (Millipore Sigma, SLGV013SL, PVDF), resulting in the retention of the conjugates on the filter membrane. PBS buffer (150 μL) was added to the filter cartridges to elute the gold conjugates, which typically resulted in recovery of ~100 μL per cartridge. The concentrated conjugates were mixed well by pipetting up and down ten times. The conjugates (5 μL) were added first to a 384-well polystyrene plate (Thermo Fisher, 12-565-506), and then the PBS control or test antibodies (in PBS, 45 μL) were added. The samples were mixed well by pipetting up and down ten times. The absorbance spectra were measured (450–650 nm) in 1 nm increments using a Biotek Synergy Neo microplate reader (BioTek, Winooski VT). The plasmon wavelengths were calculated by fitting a second order polynomial equations to the 40 absorbance measurements surrounding the maximum absorbance values and setting the first derivatives of the polynomials to zero. The plasmon shifts were calculated as the difference in the plasmon wavelengths of the test antibodies relative to the PBS control (no human antibody).

### hFc:hFcRn KD determination using surface plasmon resonance

For SPR analysis, IgG1 antibody variants were immobilized on CM5 chip surface at a density of ~500 response units (RU) via an amine coupling. hFcRn:hβ2 m dimer (Novus Biologicals) was injected at serially diluted concentrations (1000–40 nM) in PBS, pH 5.8, at a flow rate of 30 μL/min. To examine the interaction of hFcRn and antibody variants at physiological pH 7.4, hFcRn:hβ2m dimer (Novus Biologicals) was immobilized on the BIACore Chip at three different densities, 500 RU, 2000 RU, and 4000 RU via amine coupling^11^. Serially diluted antibodies (800–50 nM) were injected in PBS, pH 7.4, at a flow rate of 30 μL/min. Dissociation constants (*K*_D_s) were determined by fitting the corresponding binding isotherms for steady-state data or by fitting the kinetics for association and dissociation employing a 1:1 Langmuir mass transfer model. In addition, to detect the function of pH for hFc:hFcRn interaction, 800 nM of each antibody variants was injected under diverse pH conditions (pH 6.0–8.0) and RU_max_ was measured.

### ELISA assays

Fifty microliters of 2 μg/mL antibody variant was diluted in PBS (pH 7.4) and used to coat a 96-well polystyrene ELISA plate (Corning, Corning, NY) overnight at 4 °C. After blocking with PBS (pH 7.4) with 3% bovine serum albumin (BSA) for 1 h at room temperature, the plate was washed four times with PBS containing 0.05% Tween-20 (PBST) and incubated with serially diluted GST-hFcRn:hβ2m, His-tagged hFcγRI, GST-tagged hFcγRIIa, GST-tagged hFcγRIIb, or GST-tagged hFcγRIIIa in PBS (pH 7.4) at room temperature for 1 h. After washing four times with PBST, either 1:10,000 diluted HRP conjugated anti-His or anti-GST antibody (Rockland Immunochemicals) was added, and plates were washed with PBST. Absorbance at 450 nm was measured after development with TMB substrate (Pierce Biotechnology, Rockford, IL) according to the manufacturer’s instructions.

### RF-binding assays

Fifty microliters of RF positive serum (Lee Biosolutions), obtained from three different RA patients, was coated in a 96-well polystyrene ELISA plate (Corning, NY) overnight at 4 °C. After a blocking and washing step, plates were incubated with biotinylated antibody variants for 1 h at room temperature. The binding was detected by streptavidin-HRP and TMB substrate (Thermo Fisher Scientific) according to the manufacturer’s instructions.

### ADCC assays

Her2-overexpressing SK-BR3 breast cancer cells were cultured in complete DMEM and collected by centrifugation at 300 × *g* for 5 min. The harvested SK-BR-3 cells were washed in PBS and labeled with 4 μM Calcein AM (Life Technologies, USA) in PBS at 37 °C under 5% CO_2_ for 30 min. The Calcein-loaded SK-BR-3 cells were washed twice, resuspended in RPMI medium, and seeded into a 96-well plate at 10,000 cells/well. The various concentrations of IgG variants were also added. PBMCs were isolated from human blood from healthy donors. Briefly, 50 mL of human blood was collected in heparinized vials (BD Biosciences) and mixed by gently inverting the tube several times. Twenty-five microliters of blood was layered over 25 mL of Histopaque (Sigma-Aldrich) in 50 mL conical tubes. The tubes were centrifuged at 400 × *g* for 10 min in a swing-out bucket with no brakes. The human PBMCs were aspirated in the interphase between histopaque and medium. Human PBMCs were resuspended with red blood cell (RBC) lysis buffer (155 mM NH_4_Cl, 12 mM NaHCO_3_, and 0.1 mM EDTA), and washed twice with PBS. PBMCs were transferred into a 96-well plate at 100,000 cells/well (the ratio of tumor versus PBMCs was 1:10), and the plates were incubated at 37 °C under 5% CO_2_ for 4 h. The released calcein AM was detected at excitation and emission wavelengths of 485 and 535 nm. The percent of tumor cell lysis was calculated according to the following formula:

1 $${\mathrm{{100 \times }}}\left( {{{E - S}}} \right) \times \left( {{{M - S}}} \right)^{ - 1},$$

where *E* is the fluorescence of experimental well, *S* is the fluorescence of a well containing tumor cells and complement but no antibody, and *M* is the fluorescence of a well containing tumor cells with lysis buffer (Triton X-100 at 2% v/v, SDS 1% w/v, 100 mM NaCl, and 1 mM EDTA). As an isotype control, Rituximab was used.

### C1q deposition assay

Raji cells were resuspended in 25% human serum and then incubated with the serially diluted Rituximab-Fc variants at 37 °C for 30 min. Classical complement activation reactions were then quenched with 20 volumes of ice-cold 1% BSA-PBS. The cells were pelleted, washed once, and then probed with FITC anti-C1q (Abcam) for 30 min at 4 °C. The samples were washed and analyzed by flow cytometry.

### CDC assay

For CDC assay, Raji cells were labeled with 4 μM Calcein AM (Life Technologies, USA) in PBS at 37 °C under 5% CO_2_ for 30 min and serially diluted Rituximab variants were incubated with 25% human serum and calcein AM-loaded Raji cells at 37 °C for 1 h in 96-well plates. The supernatants were collected after centrifugation at 1000 × *g* for 10 min. The fraction of lysed Raji cells was determined by the same method as used for the ADCC assays. As an isotype control, Trastuzumab was used.

### Mice

Hemizygous hFcRn transgenic mice (line 276; aka Tg276) were generated by the F1 cross of murine FcRn-deficient B6.129X1-*Fcgrttm1Dcr*/DcrJ and hFcRn transgenic line (generated by random integration of the hFcRn cDNA) B6.Cg-*Fcgrttm1Dcr* Tg (CAG-*FCGRT*) 276 Dcr/DcrJ (The Jackson Laboratory)^5,68^. Tg276 mice experiments were performed under a protocol approved by UT Austin institutional Animal Care and Use Committee (IACUC). hFcγR^KI^ mice^51^ were generated by Regeneron Pharmaceuticals Inc. to express hFcγRI, hFcγRIIa_H131_, hFcγRIIb_I232_, hFcγRIIc_stop13_, hFcγRIIIa_V158_, and hFcγRIIIb_NA2_ polymorphic variants, as described previously^51^. hFcRn^KI^ (VG1481) and hβ2m^KI^ (VG5153) were designed and generated by Regeneron Pharmaceuticals Inc. on a mixed 62.5% C57BL/6N and 37.5% 129S6/SvEv genetic background. hIgG1-heavy chain^KI^ (hIgG1^KI^) were generated on a BALB/c background in the Cogné lab. Human kappa-light chain^KI^ (*Igκ*^KI^) were generated on a C57BL/6N background in the Bruhns lab. Mice were intercrossed at Institut Pasteur (Bruhns lab) to generate Marlene (hFcRn^KI^ hβ2m^KI^ hFcγR^KI^) or Scarlett (hFcRn^KI^ hβ2m^KI^ hFcγR^KI^ hIgG1,κ^KI^) mice and used for experiments at 10–18 weeks of age. FcγR^null^ mice expressing no FcγRs were used as controls. All mouse models demonstrated normal development and breeding patterns and hFcγR expression on different cell populations from the spleen were confirmed, as described previously^51^.

Experiments using knock in mice were validated by the Comité d'Éthique en Expérimentation Animale (CETEA) #89 (Institut Pasteur, Paris, France) under #2013-0103 and by the French Ministry of Research under agreement #00513.02.

### Endogenous mouse or human IgG detection in knock-in mice

Goat anti-human IgG-Fc fragment antibodies (1 μg) or goat anti-mouse IgG-Fc fragment antibodies (0.2 μg) were diluted in carbonate buffer (pH 9.6) (Bethyl Laboratories, Montgomery, TX) and used to coat MaxiSorp 96-well ELISA plates (Nunc, ThermoFischer scientific, Waltham, MA) overnight at 4 °C. After blocking with PBS with 3% BSA for 1 h at room temperature, plates were washed three times with PBST and incubated with serially diluted wt or KI mouse serum (or human and mouse reference serum as quantification standards), at room temperature for 3 h. After washing, HRP-conjugated anti-human IgG (diluted 1:20,000; Bethyl Laboratories Inc.) or anti-mouse IgG (diluted 1:10,000; Bethyl Laboratories Inc.) were added, and plates were washed with PBST. Absorbance at 492 vs 620 nm was measured after development with the OPD substrate (Sigma Aldrich, Saint-Louis, MO).

### Immunohistochemistry

Spleens were fixed in 10% neutral-buffered formalin for 24 h and embedded in paraffin. Tissue sections (4 μm) were blocked in PBS containing 3% BSA. Sections were stained with either an anti-human FcRn antibody (4 μg/mL, Novus Biologicals) or an anti-mouse FcRn antibody (100×; R&D Systems) for 30 min at 37 °C. Primary antibodies were detected with appropriate peroxidase-conjugated secondary antibodies and DAB detection kit (<8 min, RT). All staining reactions were accompanied by a negative control that consisted of an isotype-matched irrelevant antibody. Stained slides were evaluated with a light microscope (Nikon).

### RT PCR

Total RNA was extracted from human peripheral blood mononuclear cells or murine splenocytes using NucleoSpin RNA plus kit (Macherey-Nagel) according to the manufacturer’s recommendations. cDNAs were generated at 50 °C for 60 min using random primers and SuperScript III Reverse Transcriptase (Invitrogen). The primer pairs for the human FcRn gene *FCGRT* (L28-L179) and the mouse FcRn gene *Fcgrt* (L231-R295) (*FCGRT*: 5′-CTCTCCCTCCTGTACCACCTT-3′; 5′-ATAGCAGGAAGGTGAGCTCCT-3′; *Fcgrt*: 5′-AGCTCAAGTTCCGATTCCTG-3′; 5′- GATCTGGCTGATGAATCTAGGTC-3′) were used for amplification with GoTaq G2 polymerase (Promega). Amplification was performed by 35 cycles PCR each consisting of 94 °C for 1 min, 58 °C for 1 min, and 72 °C for 1 min. At the end of the 35 cycles, samples were run for an additional 10 min at 72 °C and analyzed by 1.5% agarose gel electrophoresis.

### Pharmacokinetics studies in mice

Animals were administered a bolus intravenous (i.v.) dose of 2 mg/kg antibody on day 0 by tail vein injection. Blood samples were obtained from the tail vein using capillary pipettes at different time points.

Serum concentration of antibody in Tg276, Marlene, or Scarlett was determined using a quantitative ELISA as follows: 96-well plates were coated with 2 μg of Goat anti-human F(ab′)_2_ fragment-specific F(ab′)_2_ (for Tg276; Jackson Immunoresearch) or 1 μg of each antigens (for Marlene and Scarlett). Her2 extracellular domain protein (Antibodies-Online) for trastuzumab, CD20 linear peptide (142–184, extracellular domain; Alpha Diagnostic Intl., Inc.) for rituximab, or IgE protein for omalizumab was used. Plates were blocked with 1% Fish gelatin (AMRESCO Inc) in PBS for 1 h, and then incubated with appropriately diluted serum samples (1:200 for earlier time points and 1:50 or 1:100 for later time points). Anti-kappa light chain antibody-HRP (Abcam) was used to detect the human antibody (dilution 1:5,000). Absorbance at 450 nm was measured after development with TMB substrate (Pierce Biotechnology, Rockford, IL) according to the manufacturer’s directions. Standard curves were generated for each antibody variant diluted into 1:100 pre-bleed mouse serum. The linear portions of standard curves generated in Prism (GraphPad Software) were then used to quantify human IgG in the serum samples.

Pharmacokinetic parameters were analyzed with a two-compartment elimination model. AUC_inf_ (area under the curve to infinity) was calculated using the log-linear trapezoidal method^20^. Terminal half-life (*T*_1/2_) was calculated using two-phase clearance models. Log-linear regression of the concentration data including at least the last six sampling time-points with measurable concentrations. Serum clearance was estimated as

2 $${\mathrm{{CL}}} = {\mathrm{{DoseAUC}}}_{\mathrm{{inf}}}^{ - 1}.$$

Volumes of distribution at steady state were calculated using the following equation:

3 $$V_{ss} = {\mathrm{{Dose}}} \times {\mathrm{{AUMC}}}_{\mathrm{{inf}}} \times (\mathrm {AUC}_{\mathrm{{inf}}})^{ - 2}.$$

### Computational modeling

All analysis was implemented in R and Stan, and can be found at [https://github.com/meyer-lab/FcRn-trafficking], release 1.0 (10.5281/zenodo.3474026). Test conditions were identified throughout to ensure model accuracy.

Exogenous IgG trafficking was modeled according to the following relationships, consistent with the graphic presented in Supplementary Fig. 10. Exogenous IgG is modeled to exchange between three compartments representing a central extracellular, peripheral extracellular, and endosomal space. The central compartment is modeled as

4 $$\frac{{\delta C_{\mathrm{c}}}}{{\delta t}} = Q(C_{\mathrm{p}} - C_{\mathrm{c}}),$$

where $C_{\mathrm{c}}$, $C_{\mathrm{p}}$, and *C*_e_ indicate the central, peripheral, and endosomal compartment concentrations, respectively, in units of μg/mL. *Q* indicates the transport rate between these two compartments in units of the central compartment volume per hour. As the entire model scales proportionally, the central compartment volume (*V*_c_) was assumed to equal 1, and all volumes are specified in relative amounts. The peripheral compartment concentration was specified as

5 $$\frac{{\delta C_{\mathrm{p}}}}{{\delta t}}V_{\mathrm{p}} = QC_{\mathrm{c}} - QC_{\mathrm{p}} - Q_{\mathrm{u}}C_{\mathrm{p}} + Q_{\mathrm{u}}C_{\mathrm{e}}f_{\mathrm{{sort}}}f_{\mathrm{{release}}},$$

where *V*_p_ and *V*_e_ are the peripheral and endosomal volumes (relative to the central compartment volume), and *Q*_u_ is the rate of uptake into cells in units of central compartment volume per hour.

*f*_sort_ indicates the fraction of endosomal IgG that is recycled, and *f*_release_ indicates the fraction of IgG presented to the cell surface that is released (as opposed to endocytosed again).

6 $$\frac{{\delta C_{\mathrm{e}}}}{{\delta t}}V_{\mathrm{e}} = Q_{\mathrm{u}}\left[ {C_{\mathrm{p}} + C_{\mathrm{e}}\left[ {(1 - f_{\mathrm{{release}}})f_{\mathrm{{sort}}} - 1} \right]} \right].$$

Implicit in this model are a few assumptions: First, there is no clearance outside of cellular uptake and lysosomal degradation. Each sorting fraction and model parameter is assumed to not vary with the concentration of exogenous IgG, therefore assuming that the modeled processes are not saturable. Finally, sorting and release are assumed to vary in the same order as their measured affinities at pH 5.8 and 7.4, with IgG of no measurable affinity at 7.4 fully released (*f*_release_ = 1).

The resultant linear ODE model was solved using the matrix exponential of the Jacobian. The half-life was found through root finding with Newton’s method. Model fitting was performed using Markov Chain Monte Carlo within Stan^69^. Priors on *V*_p_, *Q*, and *V*_*i*_*n* were all specified as log-normal distributions with a mean and deviation of 1 in their respective units. The prior for *Q*_u_ was specified as a log-normal distribution with mean *e*^0.1^ and deviation of 0.5. Sorting parameter priors were specified to be flat and constrained to be in the order dictated by their relative affinity measurements. For example, if species A had a higher endosomal affinity than B, then *f*_sort,A_ was given an even prior from 0 to 1, and _sort,B_ was given an even prior from 0 to *f*_sort,A_. The half-life of each species was compared to a normal distribution representing the average and standard error of the in vivo experimental measurements. Sampling convergence was verified through autocorrelation analysis and the Geweke criterion.

### Reporting summary

Further information on research design is available in the Nature Research Reporting Summary linked to this article.

## Supplementary information

Supplementary Information

Peer review information

Reporting Summary

## Data Availability

All the other data that support the findings of this study are available from the corresponding author upon request.

## Code availability

All analysis for computational modeling was implemented in R v3.5.3 and rstan v2.18.2, and can be found at [https://github.com/meyer-lab/FcRn-trafficking], release 1.1 (10.5281/zenodo.3474026). The code is available for access under the MIT license without restrictions.
