# Publisher Correction: An engineered human Fc domain that behaves like a pH-toggle switch for ultra-long circulation persistence

**DOI:** 10.1038/s41467-019-13458-x

**Published:** 2019-11-26

**Authors:** Chang-Han Lee, Tae Hyun Kang, Ophélie Godon, Makiko Watanabe, George Delidakis, Caitlin M. Gillis, Delphine Sterlin, David Hardy, Michel Cogné, Lynn E. Macdonald, Andrew J. Murphy, Naxin Tu, Jiwon Lee, Jonathan R. McDaniel, Emily Makowski, Peter M. Tessier, Aaron S. Meyer, Pierre Bruhns, George Georgiou

**Affiliations:** 1 Department of Chemical Engineering, University of Texas at Austin, Austin, TX USA; 2 Unit of Antibodies in Therapy and Pathology, Institut Pasteur, UMR1222 INSERMF-75015, Paris, France; 3 Experimental Neuropathology Unit, Infection and Epidemiology Department, Institut Pasteur, 25, rue du Docteur Roux, 75015 Paris, France; 4 Limoges University, Limoges, France; 5 Regeneron Pharmaceuticals, Inc., Tarrytown, NY USA; 6 Thayer School of Engineering, Dartmouth College, Hanover, NH USA; 7 Department of Pharmaceutical Sciences, University of Michigan, Ann Arbor, MI USA; 8 Department of Chemical Engineering, University of Michigan, Ann Arbor, MI USA; 9 Department of Biomedical Engineering, University of Michigan, Ann Arbor, MI USA; 10 Department of Bioengineering, University of California at Los Angeles, Los Angeles, CA USA; 11 Department of Molecular Bioscience, University of Texas at Austin, Austin, TX USA; 12 Department of Biomedical Engineering, University of Texas at Austin, Austin, TX USA; 13 Present Address: Department of Applied Chemistry, Kookmin University, Seoul, Republic of Korea

**Keywords:** Genetic engineering, Protein design

Correction to: *Nature Communications* 10.1038/s41467-019-13108-2, published online 06 November 2019.

The original version of this Article contained an error in Fig. 1d, in which all trend lines were displaced to the right. This has been corrected in both the PDF and HTML versions of the Article.

