# Supplementary material for: An engineered human Fc domain that behaves like a pH-toggle switch for ultra-long circulation persistence
Source: Nat Commun. 2019 Nov 6;10:5031. doi: 10.1038/s41467-019-13108-2 (PMC6834678; doi:10.1038/s41467-019-13108-2)
Supplement: Supplementary file 1 — Supplementary Information [file 41467_2019_13108_MOESM1_ESM.pdf]

Supplementary information

An Engineered Human Fc Domain that Behaves like a pH-Toggle Switch for Ultra-Long Circulation Persistence

C.-H. Lee et al.

a

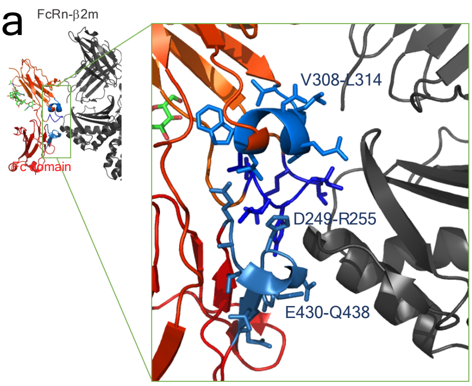

b

Human	codon	Possible residues
Asp249	VRK	Asp/ Glu/Gly/His/Lys/Asn/Gln/Arg/Ser
Thr250	VMB	Ala/Asp/Glu/His/Lys/Asn/Pro/Gln/Thr
Leu251	CDC	His/Leu/Arg
Met252	VWB	Asp/Glu/His/Ile/Lys/Leu/Met/Asn/Gln/Val
Ile253	VWH	Asp/Glu/His/Ile/Lys/Leu/Asn/Gln/Val
Ser254	VRK	Asp/ Glu/Gly/His/Lys/Asn/Gln/Arg/Ser
Arg255	VRK	Asp/ Glu/Gly/His/Lys/Asn/Gln/Arg/Ser
Val308	SWB	Asp/Glu/His/Leu/Gln/Val
Leu309	VRK	Asp/ Glu/Gly/His/Lys/Asn/Gln/Arg/Ser
Gln311	VRK	Asp/ Glu/Gly/His/Lys/Asn/Gln/Arg/Ser
Asp312	VRK	Asp/ Glu/Gly/His/Lys/Asn/Gln/Arg/Ser
Leu314	CDC	His/Leu/Arg
Glu430	VRK	Asp/ Glu/Gly/His/Lys/Asn/Gln/Arg/Ser
Leu432	CDC	His/Leu/Arg
Asn434	VRK	Asp/ Glu/Gly/His/Lys/Asn/Gln/Arg/Ser
Tyr436	BAC	Asp/His/Tyr
Gln438	VRK	Asp/ Glu/Gly/His/Lys/Asn/Gln/Arg/Ser

c

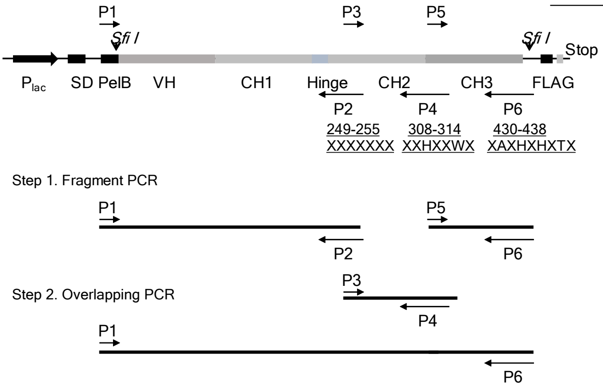

d

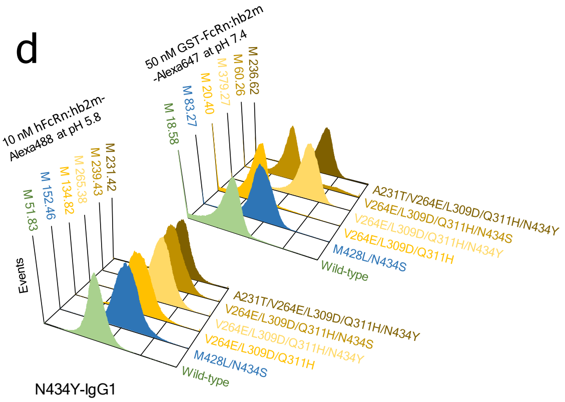

e

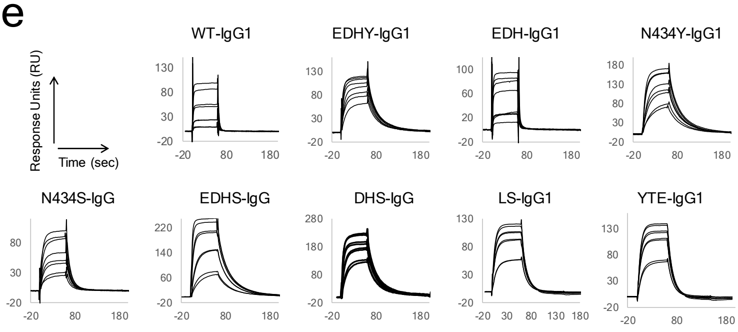

f

	K_{on} (10^5 M^{-1} sec^{-1})	K_{off} (10^{-2} sec^{-1})	K_D (nM)
WT-IgG1	5.4 ± 0.30	29 ± 0.85	550 ± 46
EDH-IgG1	4.65 ± 0.38	23 ± 0.01	487 ± 35
EDHY-IgG1	14 ± 2.0	3.8 ± 0.11	28 ± 4.7
N434Y-IgG1	7.4 ± 2.5	3.1 ± 0.11	43.6 ± 13
EDHS-IgG1	12 ± 1.3	11 ± 1.0	93 ± 1.3
N434S-IgG1	4.6 ± 2.2	8.8 ± 0.25	220 ± 110

g

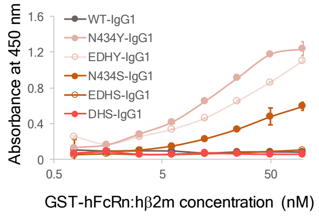

h

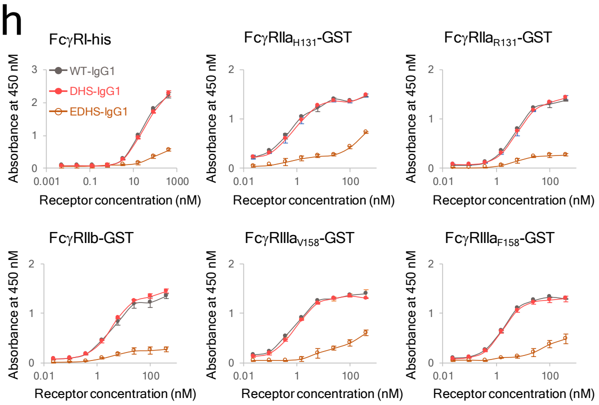

Supplementary Fig. 1 Isolation of DHS-Fc and biochemical characterization. (**a**) Binding sites of human IgG1-Fc domain for hFcRn. Residues in the Fc domain in contact with or neighboring hFcRn (< 7 Å) are highlighted in blue (hFcRn-hFc complex structure, PDB ID: 4N0U)[1]: IgG1-Fc domain (Red), hFcRn:hβ2m (Grey). (**b**) Degenerate codon scheme for the construction of the focused 17 position library to introduce His, wt, or charged amino acids. (**c**) Schematic illustration of the construction of the focused mutagenized libraries of Fc domains. (**d**) Fluorescent histogram of isolated IgG variants binding to 10 nM of hFcRn:hβ2m-Alexa488 (left) or to 50 nM of GST-hFcRn:hβ2m-Alexa647 (right). (**e**) SPR analysis of the interaction of antibody variants with hFcRn:hβ2m at pH 5.8. (**f**) Kinetic values of IgG variants for hFcRn:hβ2m at pH 5.8. Data are presented as mean ± standard deviation from three independent experiments. (**g**) ELISA analysis of antibody variants binding to GST- hFcRn:hβ2m at pH 7.4. Error bars: standard deviations from triplicate experiments. (**h**) ELISA analysis of DHS binding to his-tagged FcγRI, GST-tagged FcγRIIa$_{H131}$, GST-tagged FcγRIIa$_{R131}$, GST-tagged FcγRIIb, GST-tagged FcγRIIIa$_{V158}$, or GST-tagged FcγRIIIa$_{F158}$. Error bars: standard deviations from triplicate experiments.

a

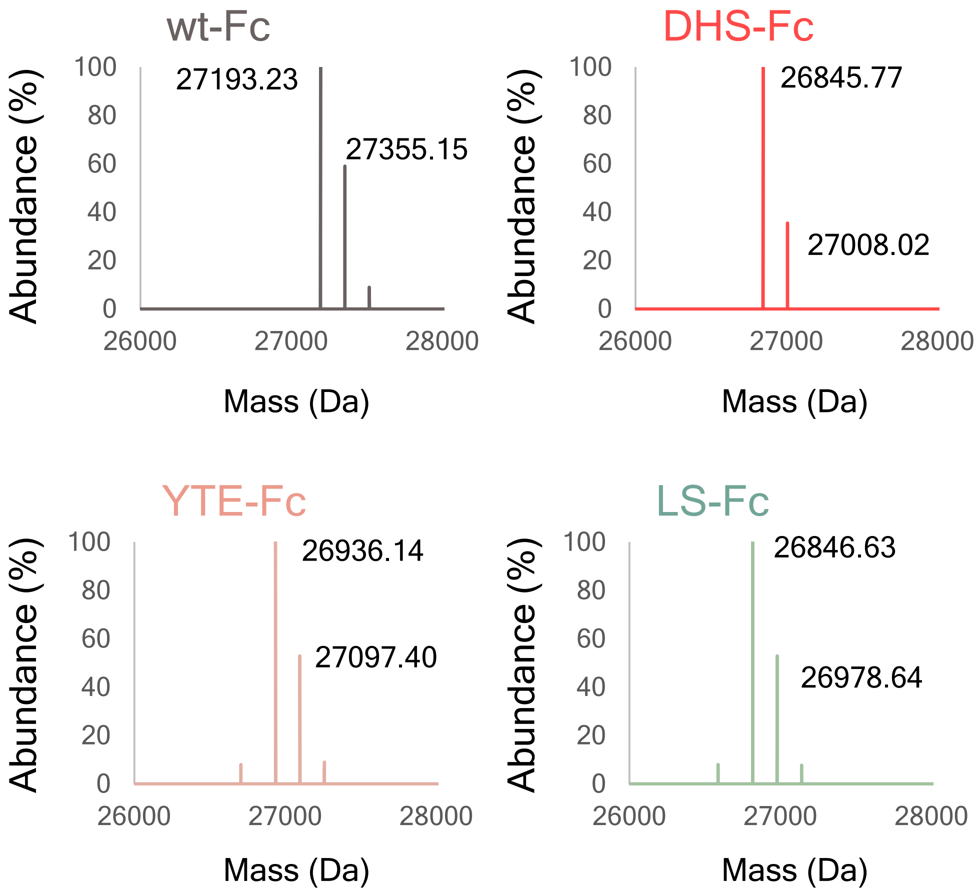

b

Fc domain	N-Glycan	Mass of Fc	Mass of glycan
wt-Fc	G0F	27193.23	1446.09
	G1F	27355.15	1608.01
DHS-Fc	G0F	26845.77	1445.97
	G1F	27008.02	1608.22
YTE-Fc	G0F	26936.14	1446.23
	G1F	27097.4	1607.49
LS-Fc	G1	26846.63	1475.82
	G1F	26978.64	1607.83

Supplementary Figure 2. LC MS/MS spectra of Fc variants. (**a**) Purified wt Fc, DHS-Fc, YTE-Fc, or LS-Fc was examined with 10 mM of dithiothreitol (DTT). Molecular weight (M.W.) estimated from the LS-MS data is shown and multiple predominant species detected are a consequence of glycan heterogeneity. (**b**) Species of N-glycan were identified by calculated mass of N-glycan using following equation: Mass of N-glycan=Detected mass of Fc domain – theoretical mass of Fc domain without N-glycan.

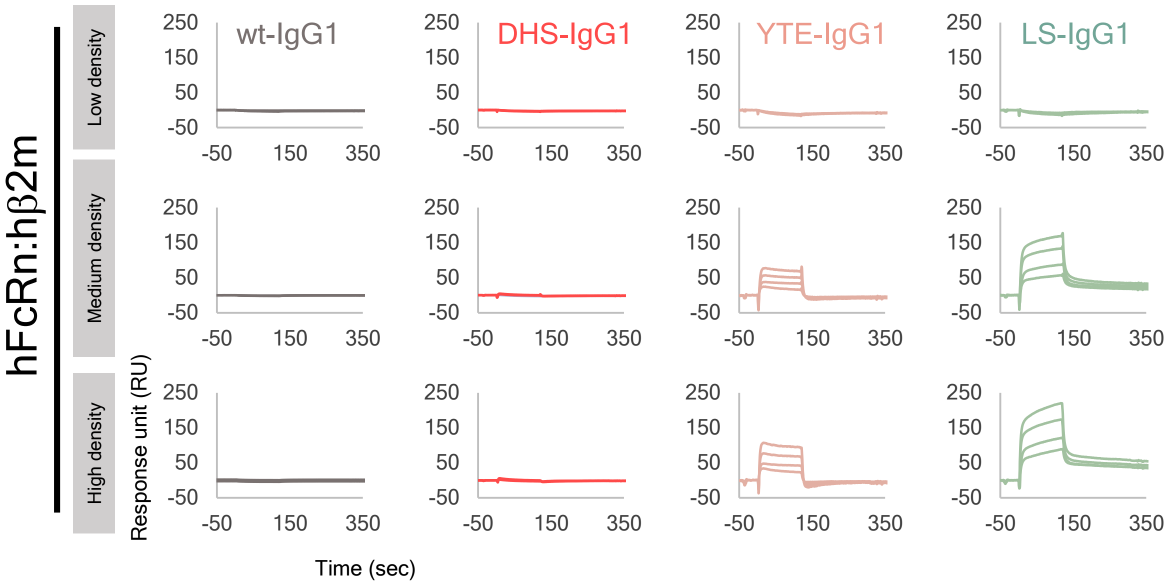

Supplementary Figure 3. Kinetic binding analysis of DHS-IgG1. SPR analysis of IgG variants (800-50 nM) with three different densities of hFcRn:hβ2m at pH 7.4. hFcRn:hβ2m was immobilized on the CM5 chips with three different levels, 500, 2000, and 4000 RU. In all sensorgrams, x-axis is time (sec) and y-axis is RU (response unit). All experiments were repeated three times independently and kinetic values are presented in **Fig. 1d.**

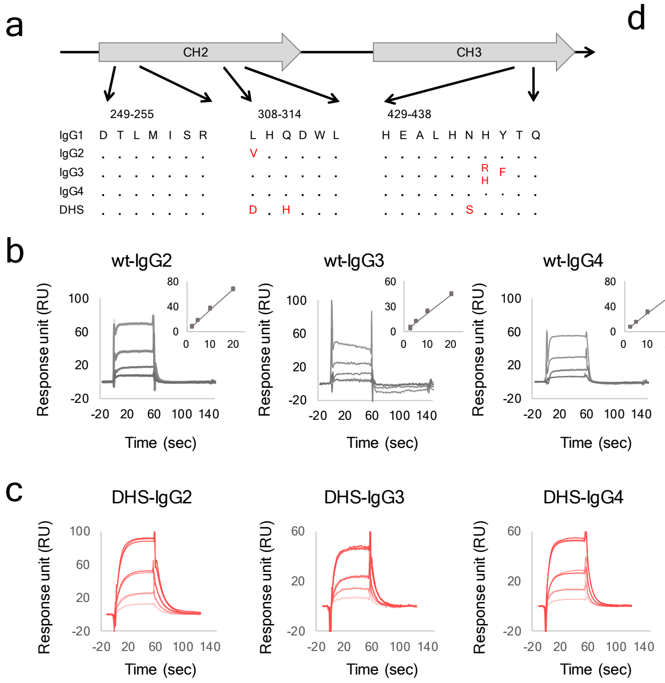

d

	K_{on} (10^5 M^{-1} sec^{-1})	K_{off} (10^{-2} sec^{-1})	K_D (nM)
wt-IgG2			2140 ± 380
DHS-IgG2	2.27 ± 0.13	8.6 ± 0.1	377 ± 26
wt-IgG3			4080 ± 790
DHS-IgG3	1.7 ± 0.13	12.6 ± 0.3	739 ± 75
wt-IgG4			2460 ± 165
DHS-IgG4	2.81 ± 0.10	15.1 ± 0.2	832 ± 16

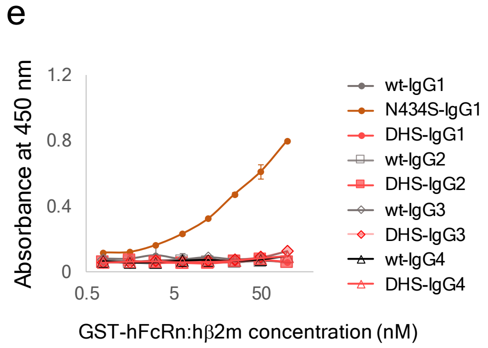

Supplementary Figure 4. Binding properties of DHS mutants of human IgG2, IgG3 and IgG4 to hFcRn:hβ2m. (**a**) Amino acids sequences of hFcRn-binding sites in DHS and wt human IgG1, IgG2, IgG3 and IgG4. (**b**-**d**) SPR analysis of the interaction of (**b**) wild type-IgG2, -IgG3 and -IgG4 and (**c**) DHS-IgG2, -IgG3 and -IgG4 with hFcRn:hβ2m at pH 5.8. All experiments were repeated three times independently and the kinetic values of each antibody are presented in (**d**). (**e**) ELISA analysis of DHS mutants of human IgG1, IgG2, IgG3 and IgG4 binding to GST-hFcRn:hβ2m at pH 7.4. Errors bars: standard deviations from triplicate experiments.

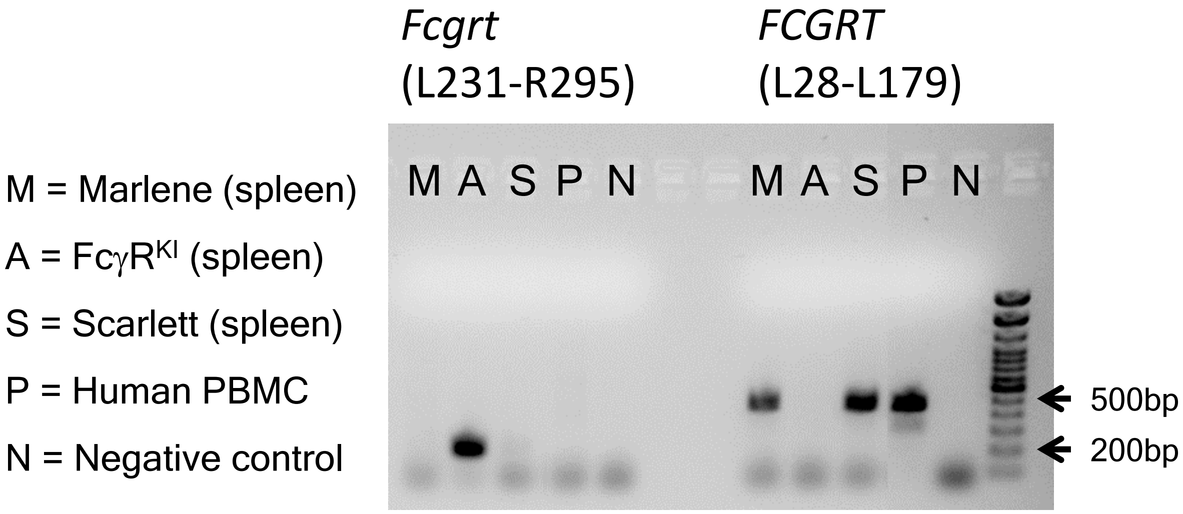

Supplementary Figure 5. RT-PCR of the FcRn encoding genes Fcgrt (mouse) and FCGRT (human) in spleen of knock-in mice. Image of an agarose gel showing the band corresponding to the 456 bp band of the FCGRT amplicon (L28-L179) and the 195 bp band of the Fcgrt amplicon (L231-R295), amplified from splenic mRNA of indicated mice or from the mRNA of human PBMCs.

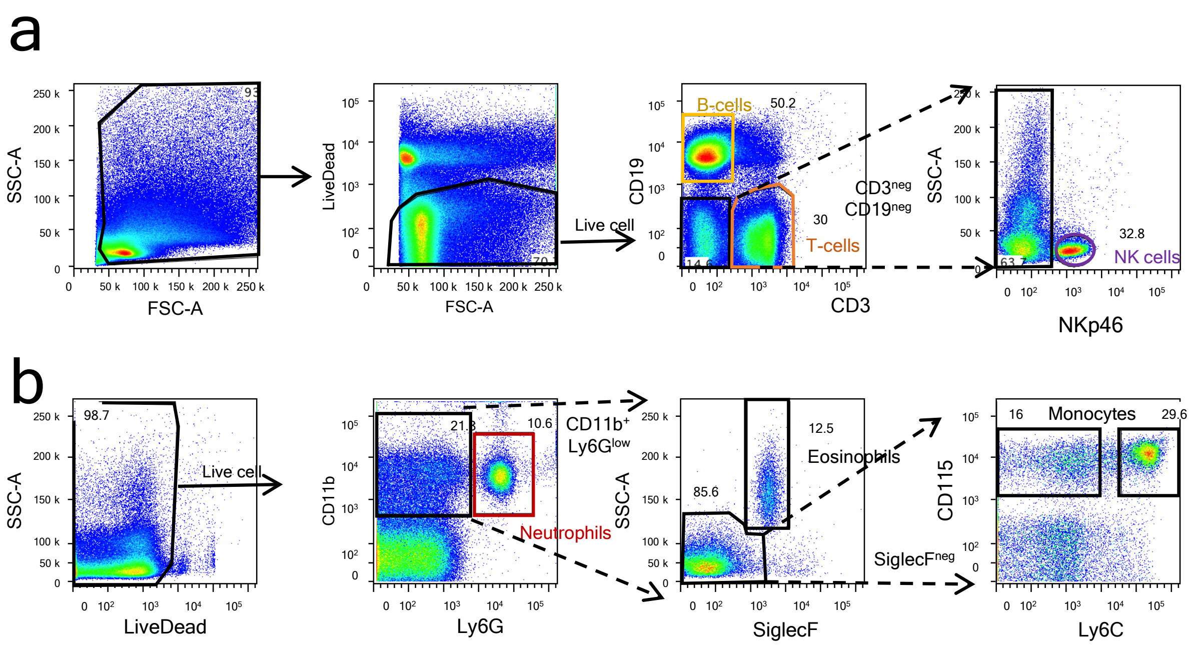

Supplementary Figure 6. Gating strategy for each immune cell population from mouse spleen or blood. (**a**) T cells (CD3^{+}), B cells (CD19^{+}) and NK Cells (CD3^{neg}, CD19^{neg}, NKp46^{+}) in the spleen; (**b**) Neutrophils (CD11b^{+}Ly6G^{hi}), Eosinophils (CD11b^{+}, Ly6G^{low}, SSC^{hi}, SiglecF^{+}), and Monocytes (CD11b^{+}, Ly6G^{low}, SiglecF^{neg}, CD115^{+}, and Ly6C$^{low\ or\ -hi}$) in the blood. Live cells were gated as DEAD-yellow negative.

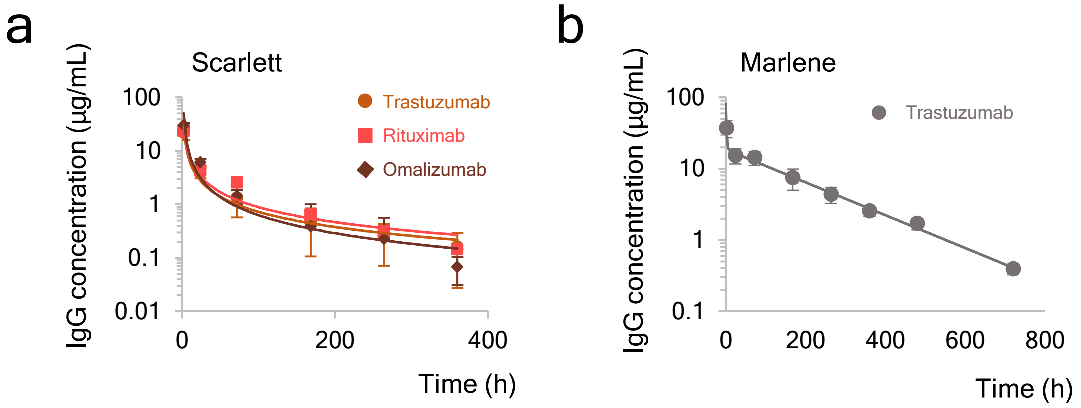

c

	Antibody	β phase $T_{1/2}$ (h)
Scarlett	Trastuzumab	92.3 ± 18.1
	Rituximab	103.5 ± 7.5
	Omalizumab	101.1 ± 6.5
Marlene	Trastuzumab	130.6 ± 4.9

Supplementary Figure 7. Pharmacokinetics of wt TrastuzumabIgG1 antibodies in Scarlett and Marlene mice. (a-b) Change in human antibody concentration in serum following tail vein administration of 2 mg/kg of antibody in Scarlett ($hFcRn^{KI}$ $h\beta2m^{KI}$ $hFc\gamma R^{KI}$ $hIgG1,\kappa^{KI}$) mice (n=7) and "Marlene" ($hFcRn^{KI}$ $h\beta2m^{KI}$ $hFc\gamma R^{KI}$) mice (n=6). (**c**) Pharmacokinetic parametersβ-phase serum half-life of antibodies is are presented as mean ± standard deviation.

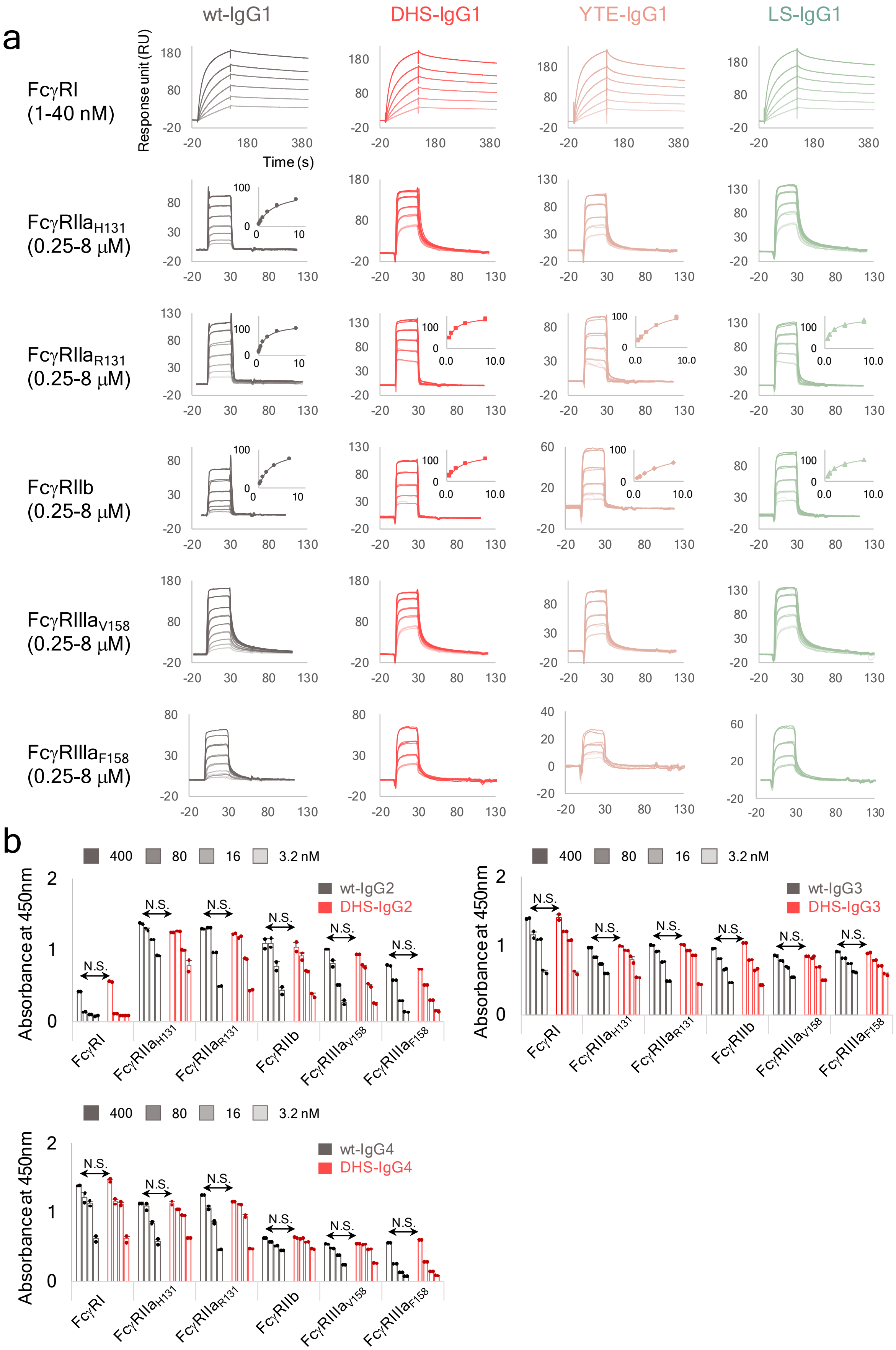

a
wt-IgG1
DHS-IgG1
YTE-IgG1
LS-IgG1
FcγRI
(1-40 nM)
FcγRIIa$_{H131}$
(0.25-8 μM)
FcγRIIa$_{R131}$
(0.25-8 μM)
FcγRIIb
(0.25-8 μM)
FcγRIIIa$_{V158}$
(0.25-8 μM)
FcγRIIIa$_{F158}$
(0.25-8 μM)
Response unit (RU)
Time (s)
b
400
80
16
3.2 nM
wt-IgG2
DHS-IgG2
wt-IgG3
DHS-IgG3
wt-IgG4
DHS-IgG4
Absorbance at 450nm
N.S.
FcγRI
FcγRIIa$_{H131}$
FcγRIIa$_{R131}$
FcγRIIb
FcγRIIIa$_{V158}$
FcγRIIIa$_{F158}$

Supplementary Figure 8. Binding analysis of DHS-IgG1, -IgG2, -IgG3 and -IgG4 to hFcγRs. (**a**) SPR analysis of antibody binding to effector FcγRs or to purified C1q. Antibody variants were immobilized on CM5 chips. The binding of His-tagged high affinity FcγRI, or of low affinity FcγRIIa, FcγRIIb, or FcγRIIIa was assayed. In all sensorgrams, x-axis is time (sec) and y-axis is RU (response unit). All experiments were repeated three times independently and kinetic values are presented in **Fig. 3a.** (**b**) ELISA analysis of DHS-IgG2, DHS-IgG3, and DHS-IgG4 binding to hFcγRs. ELISA plates were coated with antibody variants and the 400-3.2 nM of his-tagged FcγRI, GST-tagged $Fc\gamma RIIa_{H131}$, GST-tagged $Fc\gamma RIIa_{R131}$, GST-tagged FcγRIIb, GST-tagged $Fc\gamma RIIIa_{V158}$, or GST-tagged $Fc\gamma RIIIa_{F158}$ were added and the binding intensity was detected using anti-His IgG conjugated to HRP or anti-GST IgG-HRP, accordingly. P-values by two-way ANOVA test, N.S. $P \geq 0.05$. Errors bars: standard deviations from triplicate experiments.

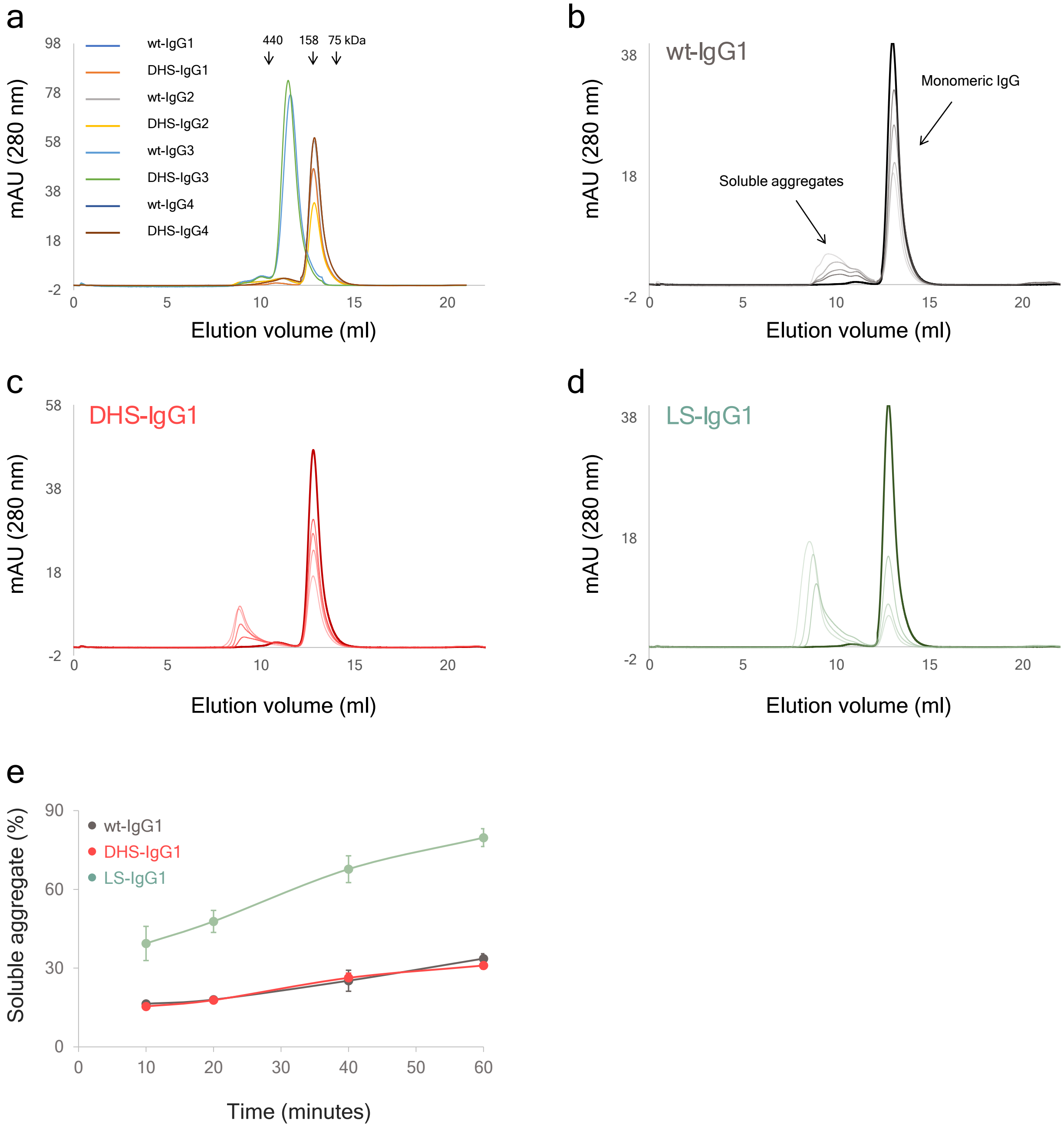

a
mAU (280 nm)
Elution volume (ml)
wt-IgG1
DHS-IgG1
wt-IgG2
DHS-IgG2
wt-IgG3
DHS-IgG3
wt-IgG4
DHS-IgG4
440
158
75 kDa
b
wt-IgG1
Monomeric IgG
Soluble aggregates
mAU (280 nm)
Elution volume (ml)
c
DHS-IgG1
mAU (280 nm)
Elution volume (ml)
d
LS-IgG1
mAU (280 nm)
Elution volume (ml)
e
Soluble aggregate (%)
Time (minutes)
wt-IgG1
DHS-IgG1
LS-IgG1

Supplementary Figure. 9. Size exclusion chromatography (SEC) analysis of antibody variants. (**a**) SEC of DHS-IgG subclasses. Each antibody variant (0.5 mg/ml) was injected into Superdex10/300 with 0.75 ml/min in pH 7.4 PBS. (**b-d**) Extent of antibody aggregation following thermal stress. Antibody variants (0.5 mg/ml) were incubated in pH 7.4 PBS at 70 ^{o}C for 0, 10, 20, 40, or 60 min (dark to bright color) and then was injected into Superdex10/300 with 0.75 ml/min in pH 7.4 PBS. Data are from one experiment representative of three experiments. Percentile of soluble aggregates of antibodies (**b-d**) was presented. Error bars: S.D. from four repeated experiments. *Note:* Trastuzumab-YTE is not shown as >90% visibly precipitated.

a

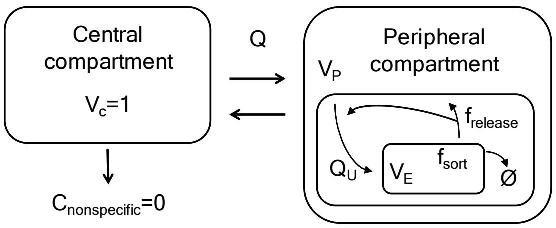

b

	Q [V_c/h]	Q_u [V_p/h]	V_{in}	V_p
Tg276	0.83<br>(0.10 - 5.97)	0.03<br>(0.02-0.04)	0.17<br>(0.04-0.45)	0.19<br>(0.05-0.46)
Scarlett	0.86<br>(0.11 - 6.51)	0.03<br>(0.02-0.04)	0.16<br>(0.04-0.42)	0.19<br>(0.05-0.45)

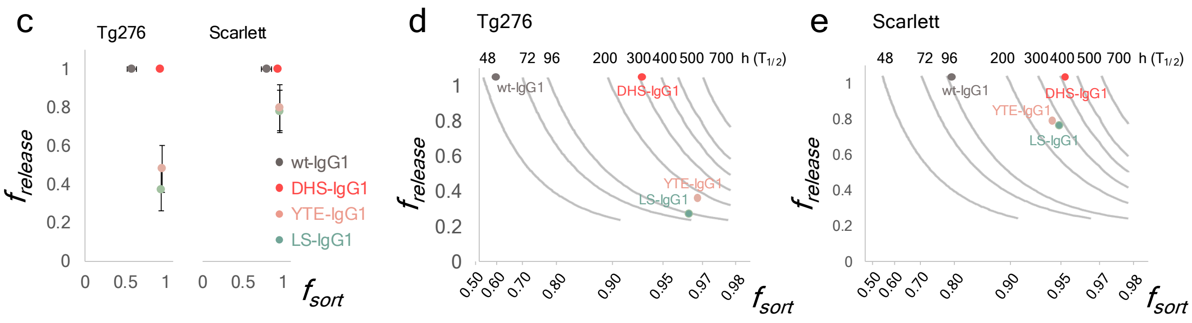

Supplementary Figure 10. The relative PK properties of each IgG mutant can be explained through a model of endosomal sorting and release. (**a**) Graphical representation of the IgG endosomal sorting model. (**b**) Fitted median values of each volumetric rate (Q, volumes of central compartment/h) and volume (V, relative to central compartment) in transport model with 95% confidence intervals. (**c**) Endosomal and surface sorting parameters for each mouse model. (**d-e**) Predicted half-life (top values) of IgG with the specified endosomal (x-axis) and surface (y-axis) sorting fractions based on the fit model; (**d**) for Tg276 and (**e**) for Scarlett. The real PK properties of each antibody variant are presented in each plot. Bars indicate 95% confidence intervals and median quantile values of each model parameter.

Supplementary Table 1. Primer list

Primer Name	Primer nucleotide sequence (5′→ 3′)
P1	GTTATTACTCGCGGCCCAGCCG
P2	CCA CGC ATG TGA CCT CAG GGG TMY BMY BDW BVW GGB GVK BMY BC T TGG GTT TTG GGG GGA AGA GGA A
P3	ACC CCT GAG GTC ACA TGC GTG G
P4	GAC CTT GCA CTT GTA CTC CTT GCC ATT GHG CCA MYB MYB GTG MYB VWS GGT GAG GAC GCT GAC CAC ACG
P5	AAT GGC AAG GAG TAC AAG TGC AAG GTC
P6	CGG GGA CAG GGA GAG GCT CTT MYB CGT GTV GTG MYB GTG GHG AG C MYB ATG CAT CAC GGA GCA TGA GAA GAC GTT
PCH017	GGCACGGTGGGCATGTGTGAGTTTTGTC
PCH021	CGGCCGCGAATTCGGCCCC

Supplementary Table 2. Pharmacokinetic values of antibody variants

	$AUC_{inf.}$ (μg*days/ml)	Clearance (ml/day/kg)	β phase $T_{1/2}$ (h)	Vss (ml/kg)
**WT-IgG2**	26.0 ± 2.0	0.38 ± 0.06	76.2 ± 7.5	0.97 ± 0.11
**DHS-IgG2**	76.3 ± 3.4	0.13 ± 0.03	188.7 ± 4.9	0.49 ± 0.02
**WT-IgG3**	38.9 ± 5.5	0.46 ± 0.02	72.6 ± 5.9	1.16 ± 0.06
**DHS-IgG3**	125.9 ± 7.3	0.16 ± 0.02	264.9 ± 42.5	0.68 ± 0.05
**WT-IgG4**	26.3 ± 3.0	0.66 ± 0.03	70.5 ± 8.7	1.73 ± 0.19
**DHS-IgG4**	57.5 ± 3.7	0.28 ± 0.05	197.0 ± 18.8	0.98 ± 0.03
