# Supplementary material for: An engineered human Fc domain that behaves like a pH-toggle switch for ultra-long circulation persistence
Source: Nat Commun. 2019 Nov 6;10:5031. doi: 10.1038/s41467-019-13108-2 (PMC6834678; doi:10.1038/s41467-019-13108-2)
Supplement: Supplementary file 2 — Peer review information [file 41467_2019_13108_MOESM2_ESM.pdf]

Editorial Note: This manuscript has been previously reviewed at another journal that is not operating a transparent peer review scheme. This document only contains reviewer comments and rebuttal letters for versions considered at Nature Communications . Mentions of the other journal have been redacted.

REVIEWERS' COMMENTS:

Reviewer #1 (Remarks to the Author):

I thank the authors for their thorough response. The revision has further improved the manuscript, which brings together a great amount of expertise and sophistication from a number of outstanding investigators. The advances presented however are mostly technical and, despite their high quality, not conceptional in nature. The pH-toggle switch mutant as well as the 9-transgene transgenic mice definitely have potential, but further validation as discussed in the reviews and rebuttal will be essential.

minor comment:

The LS mutant has been approved in the half-life extended product ravulizumab (Ultomiris) as mentioned by referee 3. The abstract only mentions clinical trials, this should be updated.

Reviewer #2 (Remarks to the Author):

I previously reviewed this manuscript when it was originally submitted to **[Redacted].** The authors have adequately addressed my comments, and I support publication of this revised manuscript in Nature Communications.

Reviewer #3 (Remarks to the Author):

The manuscript of Lee and colleagues describes the generation of a half-life extended antibody that has increased binding to FcRn at pH 5.8 and undetectable binding to FcRn at pH 7.4. The study involves elegant antibody engineering and selection techniques, and a new mouse model in which all Fc receptors (including FcRn) have been replaced by the human orthologs. The mouse strain that is used is also engineered to express human, rather than mouse, IgG. As such, the study represents a technical tour-de-force. The isolated mutant, DHS, is also improved in terms of half-life, AUC etc. over two half-life extended mutants (YTE and LS) that are currently in clinical trials. In addition, the DHS mutant retains full activity in FcgammaR and complement binding, by contrast with the YTE and LS mutants that have lost some activity. The manuscript is clearly and well written.

The authors have satisfactorily addressed the concerns that I raised during my earlier review except:

1) The KD values at pH 7.4 that I had questioned due to their similarity to the KD at pH 5.8 have now been replaced with numerically much higher numbers (Figure 1d) without any explanation. In addition, I suggested that the LS mutant might be aggregated (based on the BIAcore sensorgrams), and although it is stated that it is not, the SEC trace of the LS mutant could readily be incorporated into Figure S9d as the ‘starting’ protein before heating.

2) Concerning the new Figure S7: the word Scarlett in panel c should be positioned differently in this table so that it is clear that Trastuzumab is analyzed in this mouse strain as well as the other two antibodies.

Minor comment:
Although the readability of the text in the figures is greatly improved, some of the figure panels and corresponding text remain very hard to read due to their small size, faint text color etc.

REVIEWERS' COMMENTS:

Reviewer #1 (Remarks to the Author):

I thank the authors for their thorough response. The revision has further improved the manuscript, which brings together a great amount of expertise and sophistication from a number of outstanding investigators. The advances presented however are mostly technical and, despite their high quality, not conceptional in nature. The pH-toggle switch mutant as well as the 9-transgene transgenic mice definitely have potential, but further validation as discussed in the reviews and rebuttal will be essential.

We are grateful to the reviewer for hers/his enthusiastic evaluation of our work.

minor comment:
The LS mutant has been approved in the half-life extended product ravulizumab (Ultomiris) as mentioned by referee 3. The abstract only mentions clinical trials, this should be updated.

Thank you for your comments. We added the description of ravulizumab in the introduction (line 89-91).

Reviewer #2 (Remarks to the Author):

*I previously reviewed this manuscript when it was originally submitted to **[Redacted]**. The authors have adequately addressed my comments, and I support publication of this revised manuscript in Nature Communications.*

We thank the reviewer for hers/his comments.

Reviewer #3 (Remarks to the Author):

The manuscript of Lee and colleagues describes the generation of a half-life extended antibody that has increased binding to FcRn at pH 5.8 and undetectable binding to FcRn at pH 7.4. The study involves elegant antibody engineering and selection techniques, and a new mouse model in which all Fc receptors (including FcRn) have been replaced by the human orthologs. The mouse strain that is used is also engineered to express human, rather than mouse, IgG. As such, the study represents a technical tour-de-force. The isolated mutant, DHS, is also improved in terms of half-life, AUC etc. over two half-life extended mutants (YTE and LS) that are currently in clinical trials. In addition, the DHS mutant retains full activity in FcgammaR and complement binding, by contrast with the YTE and LS mutants that have lost some activity. The manuscript is clearly and well written.

We thank the reviewer for your comments

The authors have satisfactorily addressed the concerns that I raised during my earlier review except:

1) The KD values at pH 7.4 that I had questioned due to their similarity to the KD at pH 5.8 have now been replaced with numerically much higher numbers (Figure 1d) without any explanation. In addition, I suggested that the LS mutant might be aggregated (based on the BIAcore sensorgrams), and although it is stated that it is not, the SEC trace of the LS mutant could readily be incorporated into Figure S9d as the 'starting' protein before heating.

We apologize that there was the missing explanation. We re-analyzed the original SPR raw data using a different method. Current analysis method is the equivalent binding model.

SI Fig. 9D now contains the SEC profile of LS mutant before heating. The percentile of soluble aggregates of LS mutant before heating was approx. 2 % and thus unlikely to have accounted for the signal we observed.

2) Concerning the new Figure S7: the word Scarlett in panel c should be positioned differently in this table so that it is clear that Trastuzumab is analyzed in this mouse strain as well as the other two antibodies.

We edited SI Figure 7 as requested.

Minor comment:
Although the readability of the text in the figures is greatly improved, some of the figure panels and corresponding text remain very hard to read due to their small size, faint text color etc.

We improved the resolution of the figures.
